# Longitudinal performance development in PRO and ELITE HYROX competitions across the first seven competitive seasons

**DOI:** 10.3389/fphys.2026.1847569

**Published:** 2026-07-08

**Authors:** Ludwig Rappelt, Tim Wiedenmann, Steffen Held, Lars Heinke, Florian Micke, Pamela Wicker, Lars Donath

**Affiliations:** 1 Department of Applied Exercise Science, German Sport University Cologne, Cologne, Germany; 2 Department of Sport and Management, IST University of Applied Sciences, Düsseldorf, Germany; 3 Department of Movement and Training Science, University of Wuppertal, Wuppertal, Germany; 4 Department of Sports Science, Bielefeld University, Bielefeld, Germany

**Keywords:** concurrent training, fitness, framework, high intensity interval training, monitoring, testing

## Abstract

**Introduction:**

HYROX is a rapidly growing fitness format combining 8 km of running with 8 standardized workout stations. Despite its rapid growth, empirical evidence on performance correlates of success remains scarce. The purpose of this study is to investigate overall performance and its evolution as well as the specific contributions of the different components of individual performance in HYROX PRO and ELITE races.

**Methods:**

The analysis is based on publicly available data about race results (females: n = 11,842; males: n = 27,854) from individual PRO and ELITE competitions from the first seven seasons (2018/19-2024/25). Empirical cumulative distribution functions were used to derive season-specific percentile curves of PRO results. Changes in discipline performance were assessed for the top 100 results per season. Discipline importance was further examined using rank-based reshuffling metrics and quantile regression models. ELITE performance trends, performance convergence and Top-5 retention were additionally analyzed.

**Results:**

Between seasons 4-7, male and female PRO performances improved by ~8-10 min at the median, with larger gains at the 25^th^-75^th^ percentiles than at the 90^th^ percentile. In the Top 100, total race time improved by ~13 min (≈19%) in males and ~17min (≈21%) in females between seasons 1-7. These improvements were largely associated with faster running (males: ~8 min; females: ~10 min), which consistently accounted for ~50% of total race time and showed a low discrepancy between discipline-specific and overall rankings. Strength-determined stations exhibited greater rank reshuffling and stronger quantile-dependent effects, indicating larger absolute increases in total race time among slower performers. ELITE median performance improved from 01:06:24 to 00:57:17 h in males and from 01:11:09 to 01:03:22 h in females, while the coefficient of variation declined from >10% to <5%, indicating increasing competitive density.

**Discussion:**

HYROX has rapidly evolved into a highly competitive, sport in which overall performance is most strongly associated with running performance, alongside meaningful contributions from the strength-based stations.

## 1 Introduction

HYROX is a competitive fitness format consisting of 8 km of running, split into intervals of approximately 1 km, interspersed with eight standardized workout stations based on strength and endurance exercises within a continuous race format (Davids, 2025). Since its inception, the race has rapidly gained popularity, expanding from Germany to all six inhabited continents, thereby increasing its number of annual events tenfold (Fernández-Navarrete et al., 2025). To accommodate athletes of different performance levels, HYROX employs multiple competition categories (OPEN, PRO and ELITE) with adjusted loads as individual, double, or relay races.

As in other emerging sports, the development of athletic performance in HYROX depends on identifying and understanding the physical performance components and requirements that underpin competitive success (Plisk and Stone, 2003; Borresen and Lambert, 2009; Issurin, 2010; Read et al., 2016). These demands define which physical qualities need to be prioritized and, therefore, provide the foundation for structuring effective training programming (Turner et al., 2022). Although HYROX-specific research remains sparse, two recently published articles have examined the physiological determinants and performance-related characteristics of HYROX competitions (Brandt et al., 2025; Davids, 2025). In a review, Davids (2025) characterized the fundamental determinants of HYROX performance and proposed several potential influencing factors, including aerobic and anaerobic capacity, muscular strength and technical proficiency. Given the limited availability of empirical data, these recommendations were largely derived from available evidence from related disciplines such as CrossFit or obstacle racing. However, HYROX differs fundamentally from these formats, as CrossFit consists of constantly varied, unstandardized high-intensity workouts (Claudino et al., 2018) and obstacle racing typically combines running with terrain-based challenges, while HYROX consists of a fixed, standardized sequence of identical workout stations replicated across events worldwide. This standardization makes performance directly comparable across athletes, venues and seasons (Davids, 2025).

Brandt et al. (2025) were the first to directly assess physiological performance parameters specific to HYROX competition. Using a simulated HYROX race, they demonstrated that overall performance was positively associated with higher maximal oxygen uptake rate (V ˙O_2_max) and a greater endurance training volume, while being inversely correlated with a higher body fat content. Although these findings provide important preliminary insights, the study was limited by its sample size of only eleven recreationally trained participants (Brandt et al., 2025).

To further advance the understanding of underlying determinants for HYROX performance, larger-scale investigations including elite competitors are warranted. As the sport continues to professionalize and expand, more comprehensive datasets become available. Thus, publicly accessible race data provide an opportunity to analyze overall competition performance, discipline-specific outcomes, and the evolution of elite performance over recent years. Nevertheless, to date, no comprehensive systematic analysis has utilized these large available datasets to examine HYROX performance across different performance levels, including elite competitors. To address this issue, we conducted a large-scale analysis of publicly available race data from the first seven seasons to investigate overall performance and its evolution as well as the specific contributions of the different components of individual performance in HYROX PRO and ELITE races. The results from this analysis are expected to provide further insight into the determinants of performance and competitive success and may inform the development of future training programming and race strategies.

## 2 Materials and methods

### 2.1 Data collection process

The data of this study were obtained from the official and publicly accessible website of HYROX (hyrox.com). All individual competition data for PRO and ELITE competitions of season 1 (2018/2019) throughout season 7 (2024/2025) were extracted separately for both male and female athletes of all age groups using a multi-stage pipeline of custom and automized Python and R scripts (see **Supplementary Material 1** for the scripts). In short, stage 1 identified and listed all events per season from the website’s event overview menu, resulting in an event index containing season, event group, event identifier, and event name. In stage 2, for all individual PRO and ELITE races the base URL (https://results.hyrox.com/season-{season}/) was iteratively amended by the event identifier and retrieved the raw html-code of the paginated overview result pages for each event. During stage 3, the raw html-code was subsequently used to extract and list all links to individual race results. Thereafter, using these links the performance results were extracted into a single .xlsx-file with multiple sheets per individual race result (stage 4) and subsequently merged into single initial data sets for female athletes and male athletes (stage 5). To minimize server load, requests were throttled to one every 5-15 seconds and data collection was distributed over five weeks (29^th^ of September 2025 to 30^th^ of October 2025). No access restrictions were bypassed. The website required no registration, login, or payment to access.

**Figure 1:**
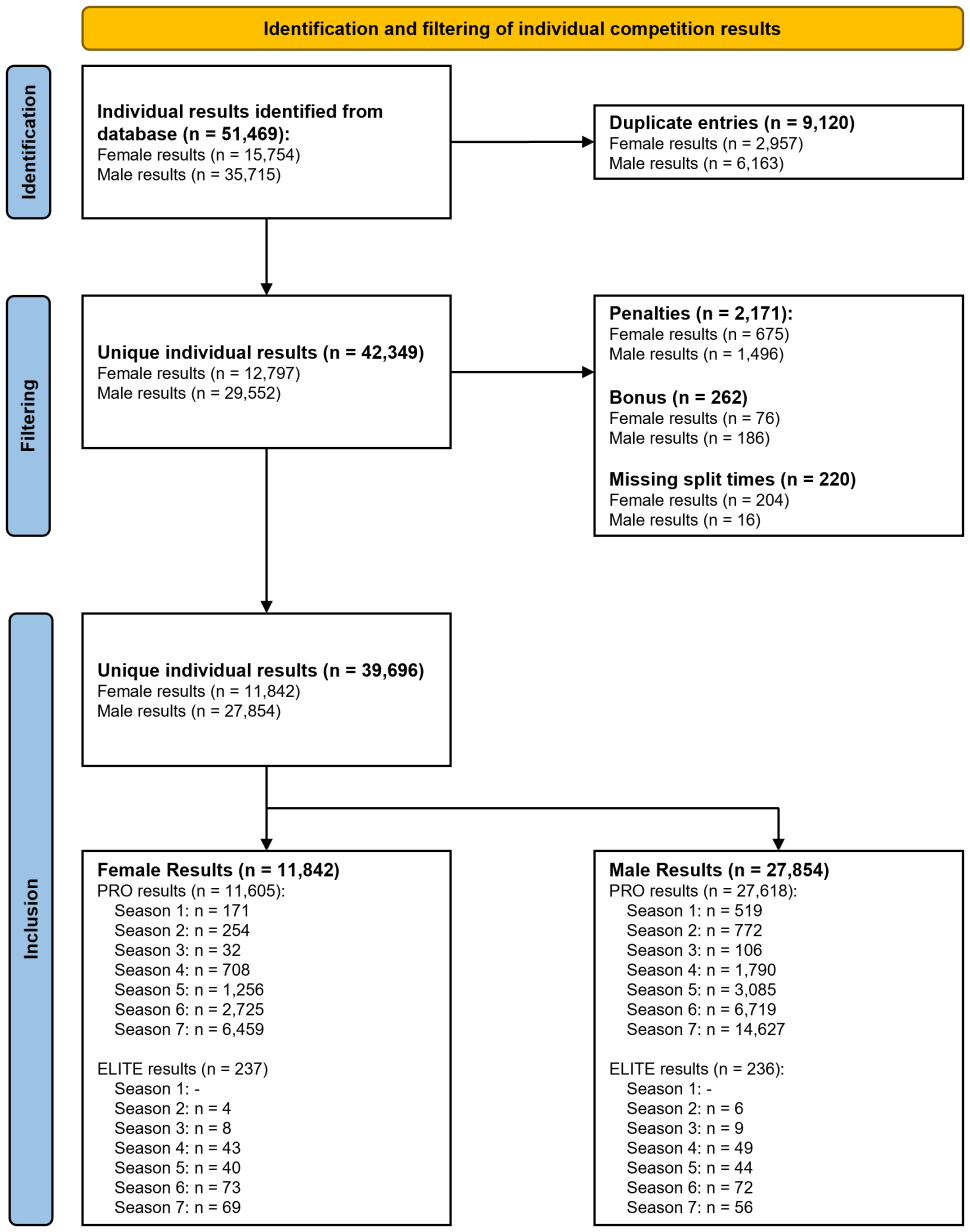
Identification and filtering process.

The initial data set included 15,754 individual results for female athletes and 35,715 for male athletes. After removing duplicates (e.g., results being listed on a specific competition day and in the overall ranking of the same event; identified by matching athlete name, event, and overall finish time in seconds), all individual results of athletes (i) having received a penalty, (ii) having had a bonus added to their times, or (iii) missing at least one discipline-specific split-time were also excluded from further analysis. Thus, a total of 11,842 female results (11,605 in PRO competitions, and 237 in ELITE competitions) as well as 27,854 male results (27,618 in PRO competitions, and 236 in ELITE competitions) were included in the empirical analysis (**Figure 1**). Manual harmonization of athlete names was only applied to ELITE competitions. For sensitivity analyses (see **Supplementary Material**), an approximate composite key (consisting of the normalized athlete name, age group, and nationality) was used in PRO results.

HYROX PRO and ELITE competitions consist globally of a consistent race format of eight running sections (total of 8 kilometer) interspersed by eight high-intensity exercise stations in a consistent order: (i) completing the virtual distance of 1000 m on the Ski ergometer (Ski-Erg), (ii) 2x25 m of pushing a weighted sled with an additional load of 152 kg (females) or 202 kg (males) (Sled-Push), (iii) 2x25 m of pulling a weighted sled with an additional load of 103 kg (females) or 153 kg (males) using a rope (Sled-Pull), (iv) completing an 80 m distance of burpees with a subsequent broad jump (Burpee Broad-Jump), (v) completing the virtual distance of 1000 m on a rowing ergometer (Rowing), (vi) carrying 2 kettlebells of 24 kg (females) or 32 kg (males) over a distance of 200 m (Farmers Carry), (vii) completing a distance of 100 m with walking lunges while carrying a sandbag of 20 kg (females) or 30 kg (males) on the shoulders (Sandbag Lunges), and (viii) completing 100 valid repetitions of performing a squat with a weighted ball of 6 kg (females) or 9 kg (males) and throwing the ball at an elevated marked target (Wallballs). While the overall running distance, exercise stations, and order of exercise stations are consistent throughout competitions, the exact distance of each running section may differ from event to event due to differences in the Rox Zone (i.e., the zone connecting the running course and the individual stations for the eight high-intensity exercise stations). Thus, we decided to only analyze the total competition time, total 8 km running time, and the individual times of the distinct high-intensity exercise stations without focusing on split times. The Running time therefore represented the cumulative time required to complete the fixed 8-km running distance and explicitly excluded time spent within the Rox Zone, which was analyzed separately.

### 2.2 Statistics

If not stated otherwise, data are presented as mean ± standard deviation (SD). To characterize performance profiles across seasons, independent empirical percentile curves were derived from each competitive season using all recorded PRO finish times. Within each season, results were ordered ascending from fastest to slowest, and percentiles ranging from the 1^st^ to 99^th^ percentile were obtained from the empirical cumulative distribution function. To compare performance distributions across seasons, predefined empirical percentiles (25^th^, 50^th^, 75^th^, and 90^th^) were compared between seasons via a non-parametric bootstrap approach. For each season contrast and percentile, resampling with replacement (5,000 iterations) was used to estimate the sampling distribution of percentile differences and compute 95% confidence intervals (CI) and two-sided *p*-values. To account for multiple testing across all season contrast and percentile levels, *p*-values were adjusted using the Holm-Bonferroni procedure. As a sensitivity analysis (**Supplementary Material**), all bootstrap comparisons were repeated restricting to one performance per athlete per season (fastest time retained).

To assess discipline-importance across seasons a combined approach was used: (i) Within each season, athletes were ranked based on their overall race time as well as separately for each discipline. For each discipline, the difference between the discipline-specific rank and overall rank was computed for each athlete and normalized to a percentage of the competitive field in each season (0% = identical ranks; 100% = maximal possible rank difference within that season). The standard deviation of these normalized rank differences was used as a metric for discipline-specific rank reshuffling, calculated separately for each season. (ii) To assess the association between discipline-specific performance and total competition time across different performance levels, univariate quantile regression models (across the 0.10, 0.25, 0.50, and 0.75 quantiles) were fitted within each season, with standardized discipline times as predictors and, to avoid the part-whole dependency between each discipline and the total competition time, the total competition time excluding the time of the discipline in focus as the dependent variable. Model fits were quantified using pseudo-R^2^, reflecting the proportional reduction in the quantile loss function relative to an intercept-only model. (iii) To identify possible differences between seasons in absolute and relative time for the whole event and all exercise stations, a linear mixed-effects model was fitted with fixed effects for season, and a random intercept for participant. For this, the respective 100 best performances per season (Top 100) were used to ensure comparable competitive levels across seasons. After an initial assessment of normal distribution using the Shapiro-Wilk-test (*p* ≥ 0.1), visual inspection using QQ-plots of residuals and verification of variance homogeneity with Levene-tests (*p* ≥ 0.1), the fixed effect was analyzed using *F*-tests (type III) with Satterthwaite approximations for the degrees of freedom. Model effect sizes are given as partial omega squared (ω_p_^2^), with 0.01 ≤ ω_p_^2^ < 0.06, 0.06 ≤ ω_p_^2^ < 0.14, ω_p_^2^ ≥ 0.14 indicating small, moderate, and large effects, respectively. Subsequently, in case of a statistically significant main effect of season, Tukey *post-hoc* tests to adjust for multiple testing were computed. To assess robustness to repeated performances (**Supplementary Material**) and event-level clustering, the Top-100 model was additionally re-estimated using a harmonized athlete key, with an added event-level random intercept, and restricting to one performance per athlete per season.

For the ELITE competition results, central tendency and dispersion of finishing times were summarized using mean values, the median, and coefficient of variation (CV) were calculated for all results and for the top 5 results per season (Top 5). 95%-CI of CV were estimated by non-parametric bootstrap (5,000 resamples).

All statistical analyses and visualizations were conducted in R (version 4.2.0) using RStudio (version 2023.06.1 + 524). For all analyses, an *α*-level of 0.05 was used as the threshold for statistical significance, unless stated otherwise. All calculations were performed with times converted into seconds. For readability, however, times are displayed as [mm:ss] or [hh:mm:ss] in tables and diagrams, if suitable.

## 3 Results

**Figure 2:**
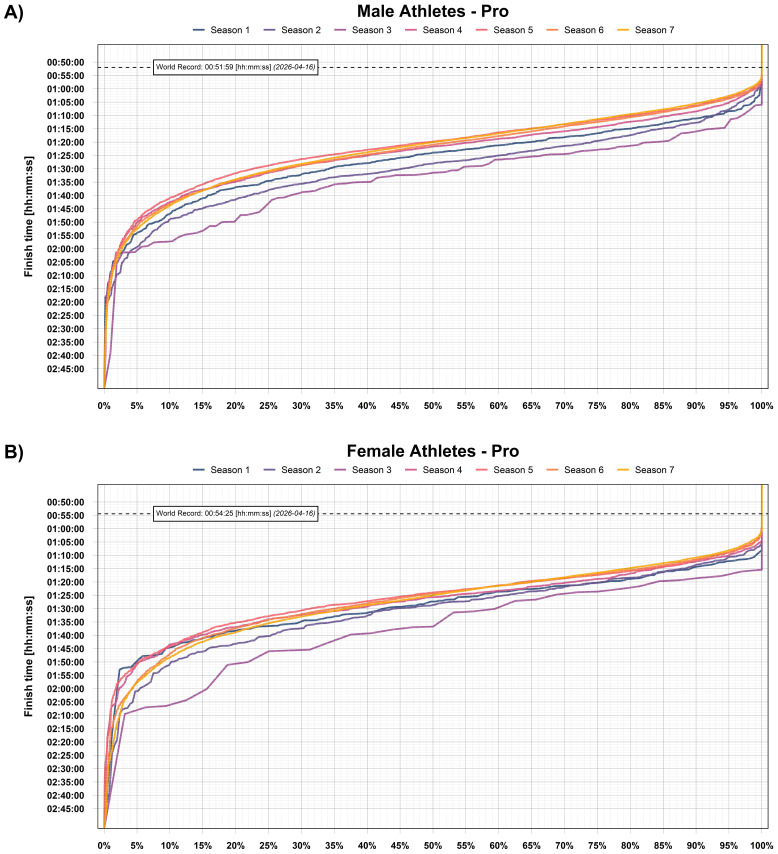
Empirical cumulative distribution functions for male **(A)** and female **(B)** PRO competition results across seasons.

Across both sexes, the full empirical percentile curves of finishing times showed most pronounced differences across the lower and central portions of the distributions, while curves converged more closely toward the upper percentiles (**Figures 2A, B**). Changes in finishing times were most consistently observed at the 25^th^ to 75^th^ percentiles, with differences at the 90^th^ percentile occurring at a lower rate; this concentration was most evident in men. Season-to-season differences at selected reference percentiles are summarized in the **Supplementary Table 2**. Sensitivity analyses restricting to one performance per athlete per season confirmed the pattern in men but yielded fewer significant contrasts in women, particularly for the small early seasons (**Supplementary Table 3**).

The normalized, discipline-specific rank reshuffling remained stable across seasons, with running consistently showing the lowest degree of rank reshuffling (≤ 20.0% in males; ≤ 16.1% in females), whereas Sled Push, Sled Pull, Ski-Erg, Farmers Carry, and Wall Balls consistently exhibited higher reshuffling values in both males and females (**Table 1**).

**Table 1:** Values represent the within-season standard deviation of normalized rank differences (discipline-specific rank minus the overall rank), expressed as percentage of the competitive field.

Discipline	Sex	Season 1	Season 2	Season 3	Season 4	Season 5	Season 6	Season 7
Running	Males	15.6	17.7	20.0	14.6	13.3	13.7	12.7
	Females	15.6	16.1	14.7	13.9	13.1	14.4	12.5
Ski-Erg	Males	23.0	22.7	22.6	25.1	21.9	22.4	21.4
	Females	24.2	23.9	20.3	23.4	23.4	22.8	21.4
Sled-Push	Males	24.8	26.6	20.7	28.4	24.3	24.0	25.3
	Females	25.8	26.0	26.9	30.0	24.4	23.5	27.1
Sled-Pull	Males	21.7	22.2	25.8	21.1	19.7	19.7	18.4
	Females	25.8	23.2	21.2	22.8	21.3	20.7	20.7
Burpee Broad-Jump	Males	20.8	22.7	26.9	20.5	18.4	20.1	18.9
	Females	19.4	21.0	22.7	19.5	20.2	20.1	18.1
Rowing	Males	18.6	18.9	22.6	19.9	17.5	18.1	16.4
	Females	23.4	20.6	16.6	20.9	19.8	19.5	18.6
Farmers Carry	Males	24.0	24.0	24.9	22.5	21.4	21.5	21.4
	Females	23.5	21.4	22.3	21.0	21.1	21.3	22.0
Sandbag Lunges	Males	18.8	19.4	25.8	19.2	18.3	18.4	17.1
	Females	19.4	16.5	22.3	17.9	17.4	17.6	16.0
Wallballs	Males	23.4	23.3	19.0	22.2	21.1	21.2	19.4
	Females	22.0	21.5	28.1	21.6	21.0	20.6	18.1

Higher values indicate greater reshuffling of athlete rankings relative to overall race outcome.

Across seasons, univariate quantile regression models showed that discipline times were positively associated with the remaining total race time at all examined quantiles (all *p* ≤ 0.001 except a small number of contrasts in Season 3) in male athletes (**Supplementary Tables 4**, **5**). After removing each discipline’s contribution to the total, pseudo-R^2^ values were highest for Rowing (0.25-0.46), followed by Sandbag Lunges (0.19-0.39), Burpee Broad-Jump (0.18-0.36), Ski-Erg (0.19-0.32), and Running (0.14-0.35), with lower values for Farmers Carry (0.10-0.33), Wallballs (0.09-0.31), Sled-Pull (0.04-0.31), and Sled-Push (0.09-0.32). A comparable pattern was observed in female athletes (**Supplementary Table 5**), with the highest independent explanatory values for Sandbag Lunges (0.17-0.46), Burpee Broad-Jump (0.17-0.40), Rowing (0.22-0.55), Farmers Carry (0.21-0.34), Running (0.14-0.37), and Ski-Erg (0.16-0.36). Lower explanatory values were found for Wallballs (0.03-0.37), Sled-Push (0.05-0.25), and Sled-Pull (0.08-0.41). Across seasons and exercise stations, coefficients were generally larger at higher quantiles (except in Season 3), indicating larger absolute changes in total time per unit increase in discipline time among slower performers. In males, the change from τ = 0.10 to τ = 0.75 (*i.e.*, Δ*β* = *β*_τ=0.75_ - *β*_τ=0.10_) ranged from Δ*β* = -146 s (Burpee Broad-Jump, Season 3) to Δ*β* = +402 s (Sled Pull, Season 7), while in females the corresponding increases ranged from Δ*β* = -74 s (Ski-Erg, Season 3) up to Δ*β* = +514 s (Sandbag Lunges, Season 2).

**Figure 3:**
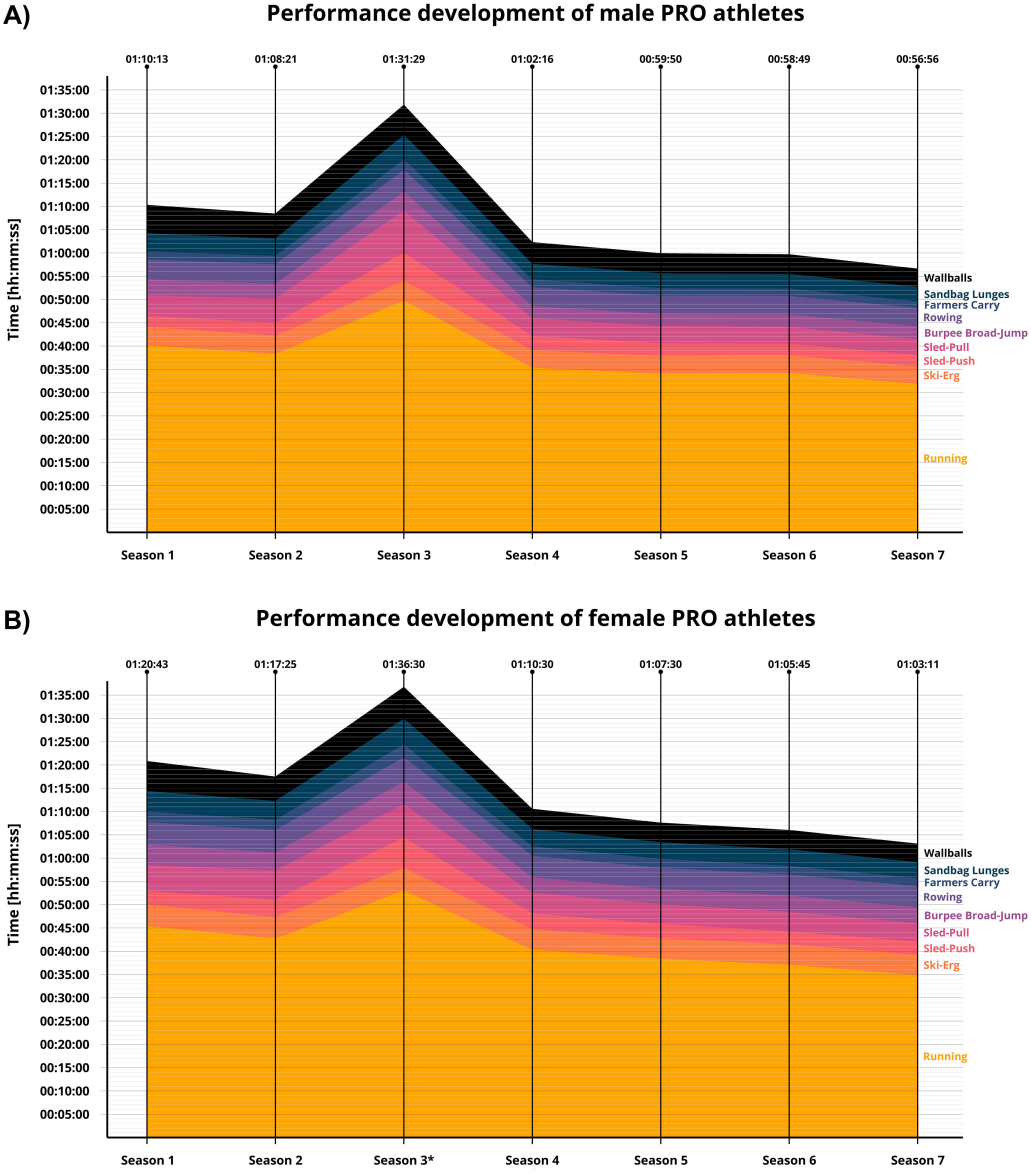
Stack plots of mean performances of the top 100 PRO results per season in males **(A)** and females **(B)**. Mean total time is indicated at the top. *Please note that for season 3 only a limited number of results were available and thus only n = 32 results are included for this season.

In both male and female PRO athletes, mean total race time of the Top 100 improved across seasons, with a pronounced deviation observed in Season 3. This deviation coincided with a substantially reduced number of available race results during that season (i.e., 32 total female results, 106 male results). From Season 4 onward, total race times again improved progressively (**Figures 3A, B**).

Reductions in total race time were largely associated with improvements in running performance, which accounted for approximately half of the total race time across seasons in both sexes. Absolute running times decreased statistically significantly from Season 1 to Season 7, while the relative contribution of running to total race time remained comparatively stable. In contrast, most non-running exercise stations (e.g., Ski-Erg, sled push/pull, rowing, and wall balls) exhibited smaller absolute time reductions but modest increases in their relative contribution to total race time over later seasons (**Tables 2**, **3**). When using a harmonized athlete key, adding an event-level random intercept, and restricting to the best performance per athlete per season in the sensitivity analyses, the absolute station and overall effects remained significant and large in both sexes, with the exception of the female Ski-Erg absolute time, which became non-significant under event clustering, and a small number of minor relative-contribution effects (Farmers Carry, Roxzone) (**Supplementary Tables 6**, **7**).

**Table 2:** Mean ± standard deviation of total race time and individual exercise stations in absolute and relative numbers for the top 100 male performances for each season.

Parameter		Season 1	Season 2	Season 3*	Season 4	Season 5	Season 6	Season 7	ANOVA
Overall time	Absolute [min:sec]	70:13 ± 03:21^b^	68:21 ± 04:23^a^	91:29 ± 13:10^a^	62:16 ± 02:17^a^	59:50 ± 01:34^a^	58:49 ± 01:14^a^	56:56 ± 00:50^a^	F(6, 352.1) = 186.8, p < 0.001; ω_p_^2^ = 0.76
	Relative [% Total]	–	–	–	–	–	–	–	–
Running	Absolute [min:sec]	35:44 ± 02:18^a^	34:00 ± 02:37^a^	42:58 ± 05:46^a^	31:34 ± 01:48^a^	30:26 ± 01:08^g^	30:04 ± 01:15^a^	27:38 ± 01:48	F(6, 414.5) = 148.5, p < 0.001; ω_p_^2^ = 0.68
	Relative [% Total]	50.9 ± 2.6^q^	49.8 ± 2.7^p^	47.3 ± 4.3^l^	50.7 ± 2.8^g^	50.9 ± 1.8^g^	51.1 ± 2.3^a^	48.5 ± 3.2	F(6, 491.0) = 20.8, p < 0.001; ω_p_^2^ = 0.19
Ski-Erg	Absolute [min:sec]	03:57 ± 00:12^b^	03:57 ± 00:11^a^	04:14 ± 00:16^a^	03:51 ± 00:08	03:47 ± 00:06	03:48 ± 00:07	03:48 ± 00:07	F(6, 485.8) = 50.3, p < 0.001; ω_p_^2^ = 0.38
	Relative [% Total]	5.6 ± 0.3^b^	5.8 ± 0.3^a^	4.7 ± 0.5^a^	6.2 ± 0.2^a^	6.3 ± 0.2^a^	6.5 ± 0.2^a^	6.7 ± 0.2^a^	F(6, 585.2) = 349.9, p < 0.001; ω_p_^2^ = 0.78
Sled-Push	Absolute [min:sec]	02:20 ± 00:26^c^	02:46 ± 00:55^d^	06:03 ± 02:08^a^	03:00 ± 00:47^a^	02:44 ± 00:24^g^	02:29 ± 00:19	02:26 ± 00:18	F(6, 437.5) = 93.5, p < 0.001; ω_p_^2^ = 0.56
	Relative [% Total]	3.3 ± 0.6^a^	4.0 ± 1.3^f^	6.5 ± 1.6^a^	4.8 ± 1.2^i^	4.6 ± 0.6	4.2 ± 0.5	4.3 ± 0.5	F(6, 544.1) = 83.9, p < 0.001; ω_p_^2^ = 0.47
Sled-Pull	Absolute [min:sec]	04:25 ± 00:51^e^	05:10 ± 01:12^a^	09:03 ± 04:32^a^	03:50 ± 00:33	03:42 ± 00:31	03:37 ± 00:23	03:27 ± 00:31	F(6, 465.1) = 57.7, p < 0.001; ω_p_^2^ = 0.42
	Relative [% Total]	6.3 ± 1.1^j^	7.5 ± 1.6^a^	9.7 ± 3.8^a^	6.1 ± 0.8	6.2 ± 0.8	6.1 ± 0.6	6.1 ± 0.9	F(6, 468.0) = 40.3, p < 0.001; ω_p_^2^ = 0.33
Burpee Broad-Jump	Absolute [min:sec]	03:26 ± 00:34^a^	03:10 ± 00:39^a^	04:03 ± 01:03^a^	02:36 ± 00:22	02:34 ± 00:22	02:39 ± 00:19	02:44 ± 00:16	F(6, 464.1) = 47.8, p < 0.001; ω_p_^2^ = 0.37
	Relative [% Total]	4.9 ± 0.7^f^	4.6 ± 0.9^k^	4.4 ± 0.9	4.2 ± 0.5^i^	4.3 ± 0.6^i^	4.5 ± 0.5	4.8 ± 0.5	F(6, 489.3) = 13.3, p < 0.001; ω_p_^2^ = 0.13
Rowing	Absolute [min:sec]	04:13 ± 00:13^b^	04:13 ± 00:21^a^	04:39 ± 00:20^a^	04:03 ± 00:08°	03:58 ± 00:06	03:59 ± 00:08	03:56 ± 00:05	F(6, 429.5) = 51.9, p < 0.001; ω_p_^2^ = 0.41
	Relative [% Total]	6.0 ± 0.3^a^	6.2 ± 0.6^a^	5.1 ± 0.5^a^	6.5 ± 0.2^a^	6.6 ± 0.2^a^	6.8 ± 0.2^a^	6.9 ± 0.1	F(6, 454.1) = 182.6, p < 0.001; ω_p_^2^ = 0.70
Farmers Carry	Absolute [min:sec]	01:48 ± 00:21^b^	01:47 ± 00:20^a^	02:16 ± 00:32^a^	01:41 ± 00:15^i^	01:36 ± 00:12^g^	01:33 ± 00:08^a^	01:25 ± 00:07	F(6, 470.7) = 50.4, p < 0.001; ω_p_^2^ = 0.38
	Relative [% Total]	2.6 ± 0.5	2.6 ± 0.5^g^	2.5 ± 0.5^m^	2.7 ± 0.4^g^	2.7 ± 0.3^g^	2.6 ± 0.2	2.5 ± 0.2	F(6, 502.0) = 4.3, p < 0.001; ω_p_^2^ = 0.04
Sandbag Lunges	Absolute [min:sec]	03:52 ± 00:34^b^	03:43 ± 00:35^a^	05:15 ± 01:24^a^	03:20 ± 00:26^i^	03:13 ± 00:20	03:10 ± 00:16	03:07 ± 00:16	F(6, 427.9) = 54.1, p < 0.001; ω_p_^2^ = 0.42
	Relative [% Total]	5.5 ± 0.8	5.4 ± 0.7	5.7 ± 1.2	5.4 ± 0.6	5.4 ± 0.5	5.4 ± 0.4	5.5 ± 0.5	F(6, 477.7) = 1.70, p = 0.119; ω_p_^2^ = 0.00
Wallballs	Absolute [min:sec]	06:06 ± 01:09^r^	05:23 ± 01:02^a^	06:31 ± 01:55^a^	04:40 ± 00:39^a^	04:18 ± 00:32^g^	04:13 ± 00:31^a^	03:55 ± 00:25	F(6, 463.4) = 63.5, p < 0.001; ω_p_^2^ = 0.44
	Relative [% Total]	8.7 ± 1.5^a^	7.9 ± 1.4^a^	7.0 ± 1.4	7.5 ± 1.0^g^	7.2 ± 0.8	7.2 ± 0.9	6.9 ± 0.7	F(6, 480.0) = 28.5, p < 0.001; ω_p_^2^ = 0.25
Roxzone	Absolute [min:sec]	04:27 ± 01:05^f^	04:19 ± 00:56^f^	06:48 ± 02:23^a^	03:44 ± 01:06	03:37 ± 00:44	04:09 ± 01:23	04:10 ± 00:58	F(6, 516.4) = 54.8, p < 0.001; ω_p_^2^ = 0.38
	Relative [% Total]	6.3 ± 1.4^n^	6.3 ± 1.2^n^	7.4 ± 2.1^k^	6.0 ± 1.7^i^	6.1 ± 1.2^i^	7.1 ± 2.4	7.3 ± 1.7	F(6, 693.0) = 12.0, p < 0.001; ω_p_^2^ = 0.09

^a^ = significantly different from all subsequent Seasons; ^b^ = significantly different from Seasons 3-7; ^c^ = significantly different from Seasons 2-4; ^d^ = significantly different from Seasons 3, 4; ^e^ = significantly different from Seasons 2, 3, 5-7; ^f^ = significantly different from Seasons 3-5; ^g^ = significantly different from Season 7; ^h^ = significantly different from Seasons 5, 6; ^i^ = significantly different from Seasons 6, 7; ^j^ = significantly different from Seasons 2, 3; ^k^ = significantly different from Seasons 4, 5; ^l^ = significantly different from Seasons 4-6; ^m^ = significantly different from Season 4; ^n^ = significantly different from Seasons 3, 6, 7; ^o^ = significantly different from Seasons 5, 7; ^p^ = significantly different from Seasons 3, 5, 6; ^q^ = significantly different from Seasons 3, 7; ^r^ = significantly different from Seasons 2, 4-7.

**Table 3:** Mean ± standard deviation of total race time and individual exercise stations in absolute and relative numbers for the Top 100 female performances for each season.

Parameter		Season 1	Season 2	Season 3*	Season 4	Season 5	Season 6	Season 7	ANOVA
Overall time	Absolute [min:sec]	80:43 ± 05:38^b^	77:25 ± 05:03^a^	96:30 ± 16:18^a^	70:30 ± 02:29^a^	67:30 ± 02:28^a^	65:45 ± 01:49^a^	63:11 ± 01:01	F(6, 363.3) = 150.9, p < 0.001; ω_p_^2^ = 0.71
	Relative [% Total]	–	–	–	–	–	–	–	–
Running	Absolute [min:sec]	40:20 ± 02:57^a^	37:42 ± 03:05^a^	45:57 ± 07:25^a^	36:09 ± 01:44^a^	34:28 ± 01:38^a^	32:55 ± 01:51^a^	30:17 ± 01:27	F(6, 389.3) = 103.3, p < 0.001; ω_p_^2^ = 0.61
	Relative [% Total]	50.0 ± 3.0^k^	48.7 ± 3.0^l^	47.8 ± 4.0^l^	51.3 ± 2.3^g^	51.1 ± 2.2^a^	50.1 ± 2.6	47.9 ± 2.1	F(6, 518.2) = 22.2, p < 0.001; ω_p_^2^ = 0.19
Ski-Erg	Absolute [min:sec]	04:39 ± 00:15^b^	04:38 ± 00:17^a^	04:51 ± 00:19^a^	04:24 ± 00:09	04:23 ± 00:09	04:23 ± 00:11	04:23 ± 00:09	F(6, 476.7) = 30.1, p < 0.001; ω_p_^2^ = 0.27
	Relative [% Total]	5.8 ± 0.4^a^	6.0 ± 0.4^a^	5.1 ± 0.6^a^	6.2 ± 0.2^a^	6.5 ± 0.2^a^	6.7 ± 0.3^a^	6.9 ± 0.3	F(6, 494.3) = 153.3, p < 0.001; ω_p_^2^ = 0.65
Sled-Push	Absolute [min:sec]	02:49 ± 00:38^c^	03:45 ± 01:11^a^	06:33 ± 02:31^a^	03:24 ± 00:47^a^	03:03 ± 00:29	02:48 ± 00:27	02:47 ± 00:29	F(6, 503.3) = 76.8, p < 0.001; ω_p_^2^ = 0.47
	Relative [% Total]	3.5 ± 0.7^a^	4.8 ± 1.5^a^	6.7 ± 2.1^i^	4.8 ± 1.1	4.5 ± 0.7	4.3 ± 0.7	4.4 ± 0.7	F(6, 549.6) = 44.4, p < 0.001; ω_p_^2^ = 0.32
Sled-Pull	Absolute [min:sec]	05:37 ± 01:19^d^	06:17 ± 01:36^e^	07:08 ± 02:15^a^	04:26 ± 00:45^f^	04:17 ± 00:38^g^	04:10 ± 00:35	03:58 ± 00:21	F(6, 478.7) = 70.1, p < 0.001; ω_p_^2^ = 0.46
	Relative [% Total]	6.9 ± 1.5^d^	8.1 ± 1.9^a^	7.3 ± 1.3^i^	6.3 ± 1.0	6.3 ± 0.9	6.3 ± 0.8	6.3 ± 0.6	F(6, 523.0) = 36.8, p < 0.001; ω_p_^2^ = 0.29
Burpee Broad-Jump	Absolute [min:sec]	04:23 ± 00:47^h^	03:52 ± 00:50^a^	04:52 ± 01:16^a^	03:20 ± 00:30	03:16 ± 00:28	03:30 ± 00:30	03:27 ± 00:23	F(6, 483.0) = 35.3, p < 0.001; ω_p_^2^ = 0.30
	Relative [% Total]	5.4 ± 0.8^n^	5.0 ± 1.0°	5.0 ± 0.8^g^	4.7 ± 0.7^i^	4.8 ± 0.6^a^	5.3 ± 0.7	5.5 ± 0.6	F(6, 524.4) = 15.6, p < 0.001; ω_p_^2^ = 0.14
Rowing	Absolute [min:sec]	04:51 ± 00:14^b^	04:49 ± 00:17^a^	05:13 ± 00:26^a^	04:37 ± 00:12^a^	04:31 ± 00:10	04:33 ± 00:10	04:30 ± 00:08	F(6, 486.0) = 61.7, p < 0.001; ω_p_^2^ = 0.42
	Relative [% Total]	6.0 ± 0.4^a^	6.2 ± 0.5^a^	5.5 ± 0.5^a^	6.6 ± 0.3^a^	6.7 ± 0.2^a^	6.9 ± 0.2^a^	7.1 ± 0.2	F(6, 515.7) = 121.8, p < 0.001; ω_p_^2^ = 0.58
Farmers Carry	Absolute [min:sec]	02:17 ± 00:33^b^	02:11 ± 00:29^a^	02:46 ± 00:45^a^	02:01 ± 00:17^i^	01:56 ± 00:17^g^	01:50 ± 00:15^a^	01:44 ± 00:10	F(6, 476.7) = 33.4, p < 0.001; ω_p_^2^ = 0.29
	Relative [% Total]	2.8 ± 0.6	2.8 ± 0.5^g^	2.9 ± 0.5	2.8 ± 0.4^g^	2.9 ± 0.4^g^	2.8 ± 0.3	2.8 ± 0.3	F(6, 515.7) = 3.1, p = 0.006; ω_p_^2^ = 0.02
Sandbag Lunges	Absolute [min:sec]	04:23 ± 00:41^a^	04:02 ± 00:36^a^	05:30 ± 01:04^a^	03:43 ± 00:28^a^	03:33 ± 00:27^g^	03:35 ± 00:19	03:26 ± 00:18	F(6, 496.2) = 70.8, p < 0.001; ω_p_^2^ = 0.45
	Relative [% Total]	5.4 ± 0.7^p^	5.2 ± 0.6^p^	5.7 ± 0.7^a^	5.3 ± 0.6	5.2 ± 0.6	5.4 ± 0.5	5.4 ± 0.5	F(6, 530.0) = 5.4, p < 0.001; ω_p_^2^ = 0.05
Wallballs	Absolute [min:sec]	06:27 ± 01:43^d^	05:14 ± 01:03^a^	06:51 ± 02:33^a^	04:20 ± 00:46^g^	04:13 ± 00:39	04:09 ± 00:35	03:59 ± 00:27	F(6, 498.1) = 56.5, p < 0.001; ω_p_^2^ = 0.40
	Relative [% Total]	8.0 ± 1.9^d^	6.8 ± 1.3	7.0 ± 1.7^a^	6.1 ± 1.0	6.2 ± 0.9	6.3 ± 0.8	6.3 ± 0.7	F(6, 531.7) = 23.1, p < 0.001; ω_p_^2^ = 0.20
Roxzone	Absolute [min:sec]	05:05 ± 01:22^b^	04:59 ± 01:03^j^	07:04 ± 02:23^a^	04:09 ± 00:54	03:56 ± 00:54^g^	04:09 ± 01:04	04:34 ± 00:54	F(6, 543.5) = 33.1, p < 0.001; ω_p_^2^ = 0.26
	Relative [% Total]	6.3 ± 1.5^q^	6.4 ± 1.2^g^	7.2 ± 1.7^a^	5.9 ± 1.2^g^	5.8 ± 1.3^g^	6.3 ± 1.6^a^	7.2 ± 1.4	F(6, 545.9) = 12.0, p < 0.001; ω_p_^2^ = 0.11

^a^ = significantly different from all subsequent Seasons; ^b^ = significantly different from Seasons 3-7; ^c^ = significantly different from Seasons 2-4; ^d^ = significantly different from Seasons 2, 4-7; ^e^ = significantly different from Seasons 4-7; ^f^ = significantly different from Season 6; ^g^ = significantly different from Season 7; ^h^ = significantly different from Seasons 2, 4-6; ^i^ = significantly different from Seasons 6, 7; ^j^ = significantly different from Seasons 3-6; ^k^ = significantly different from Seasons 2-5, 7; ^l^ = significantly different from Seasons 4-6; ^m^ = significantly different from Seasons 3, 5-7; ^n^ = significantly different from Seasons 2-5; ^o^ = significantly different from Seasons 4, 7; ^p^ = significantly different from Season 3; ^q^ = significantly different from Seasons 3, 7. *for Season 3 only 32 performances could be included due to a limited number of races in this season.

**Figure 4:**
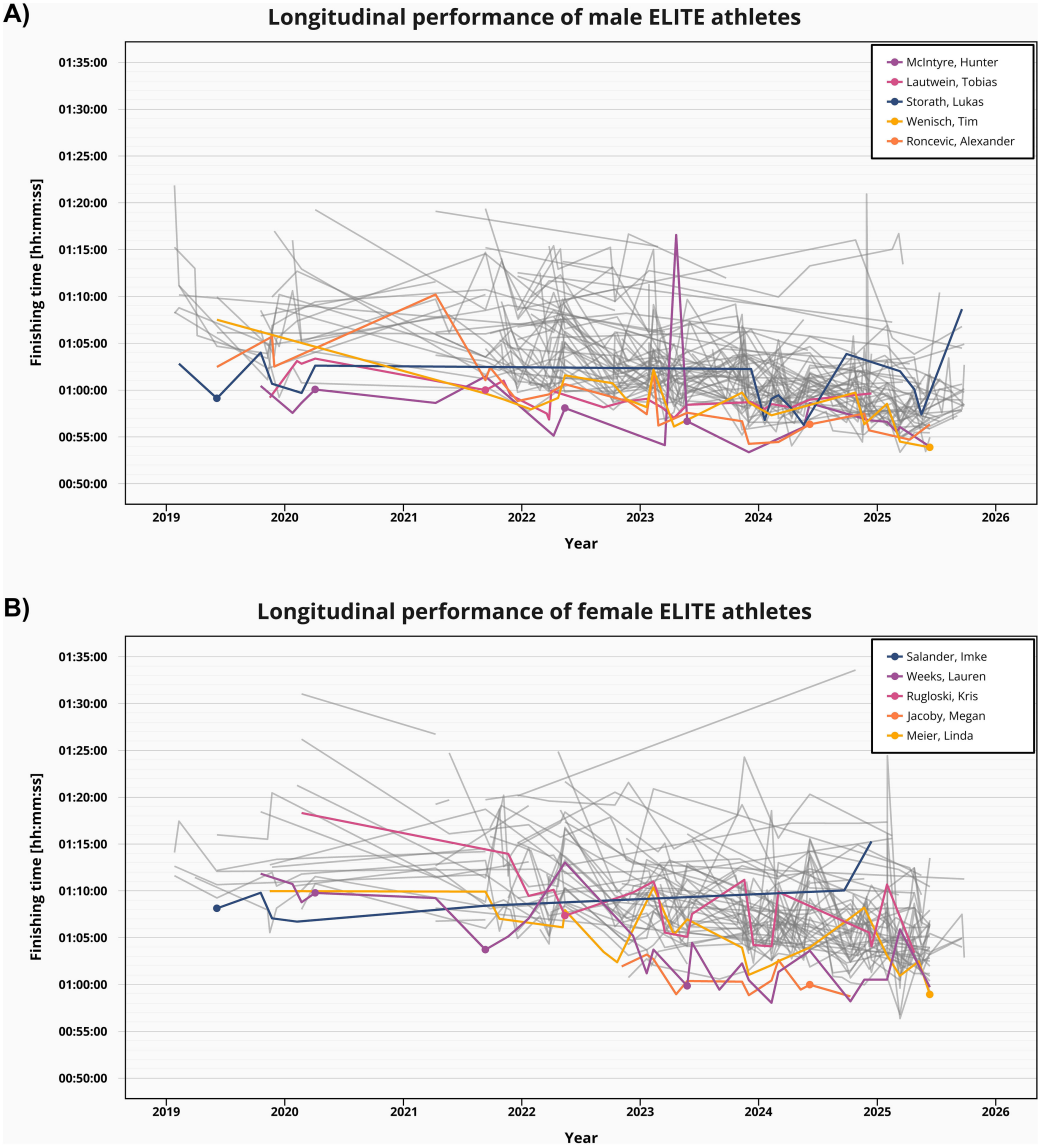
Individual performance development of **(A)** male and **(B)** female ELITE HYROX athletes. World champions are indicated in colour with their respective title-winning performances indicated as points. Please note that to improve visualization, the underlying data consists of results from ELITE races amended by occurrences of ELITE athletes in PRO events. For all other analyses regarding ELITE performances, only results from ELITE races have been used.

ELITE performances in male athletes also improved notably across seasons (**Figure 4A**), with the median finishing time decreasing from 01:06:24 h in Season 2 to 00:57:17 h in Season 7, accompanied by a substantial reduction in performance variability (overall CV 10.4% 95%CI[4.2-13.2] in Season 2 vs 4.7% 95%CI[3.5-5.7] in Season 7). Improvements were also evident at the competitive ceiling, with Top 5 median performances improving by almost 10 minutes from Season 2 (01:03:22 h) to Season 7 (00:53:58 h) (**Table 4**). Adjacent season Top 5 retention showed a considerable overlap between seasons, with one athlete retaining between Seasons 2 and 3, two athletes between Seasons 3 and 4 as well as 6 and 7, and three athletes between Seasons 4 to 6.

**Table 4:** Mean ± standard deviation and coefficients of variation (CV) of finishing times of all elite performances and top 5 elite performances per season in male athletes.

Season	Performances [n]	Overall finishing time [hh:mm:ss]	Overall CV (95%-CI) [%]	Top 5 finishing time [hh:mm:ss]	Top 5 CV (95%-CI) [%]
Season 1	–	-	-	-	–
Season 2	6	01:07:31 ± 00:07:01	10.4 (4.2 – 13.2)	01:05:10 ± 00:04:29	6.9 (1.9 – 8.1)
Season 3	9	01:10:29 ± 00:06:33	9.3 (4.1 – 12.1)	01:06:04 ± 00:05:12	7.9 (2.7 – 10.0)
Season 4	49	01:06:48 ± 00:05:48	8.7 (6.7 – 10.2)	00:59:30 ± 00:01:00	1.7 (0.3 – 2.1)
Season 5	44	01:01:48 ± 00:03:36	5.8 (3.9 – 7.5)	00:57:40 ± 00:00:42	1.2 (0.5 – 1.5)
Season 6	72	00:59:23 ± 00:02:34	4.3 (3.5 – 5.0)	00:54:37 ± 00:00:55	1.7 (0.6 – 2.2)
Season 7	56	00:57:42 ± 00:02:41	4.7 (3.5 – 5.7)	00:54:07 ± 00:00:35	1.1 (0.4 – 1.4)

95% confidence intervals (95%-CI) were obtained by non-parametric bootstrap resampling (5,000 iterations).

Similar results were found in ELITE female performances (**Figure 4B**) with the median finishing time decreasing from 01:11:09 h in Season 2 to 01:03:22 h in Season 7. Performance variability declined from Season 4 onward (overall CV: 9.5% 95%CI[6.4-12.0] in Season 4 vs. 4.8% 95%CI[4.0-5.5]). As in male performances, improvements were also evident at the competitive ceiling, with the Top 5 median performance improving substantially from Season 2 (01:11:09 h) to Season 7 (00:57:48 h) (**Table 5**). Adjacent season Top 5 retention showed a higher variability compared to males with only one (Season 2 to 3 and Season 4 to 5) or two athletes retaining between seasons (Season 3 to 4 and Season 5 throughout Season 7).

**Table 5:** Mean ± standard deviation and coefficients of variation (CV) of finishing times of all Elite performances and Top 5 Elite performances per season in female athletes.

Season	Performances [n]	Overall finishing time [hh:mm:ss]	Overall CV (95%-CI) [%]	Top 5 finishing time [hh:mm:ss]	Top 5 CV (95%-CI) [%]
Season 1	–	-	-	-	–
Season 2	4	01:11:00 ± 00:00:57	1.3 (0.3 – 1.6)	01:11:00 ± 00:00:57	1.3 (0.3 – 1.6)
Season 3	8	01:13:20 ± 00:06:37	9.0 (2.8 – 11.7)	01:09:36 ± 00:02:19	3.3 (0.9 – 4.2)
Season 4	43	01:13:54 ± 00:07:02	9.5 (6.4 – 12.0)	01:05:27 ± 00:01:12	1.8 (0.6 – 2.5)
Season 5	40	01:06:28 ± 00:04:02	6.0 (4.2 – 7.9)	01:01:08 ± 00:01:01	1.7 (0.6 – 2.0)
Season 6	73	01:06:04 ± 00:04:14	6.4 (5.1 – 7.5)	00:59:32 ± 00:01:02	1.7 (0.3 – 2.1)
Season 7	69	01:03:19 ± 00:03:02	4.8 (4.0 – 5.5)	00:57:48 ± 00:01:10	2.0 (0.4 – 2.3)

95% confidence intervals (95%-CI) were obtained by non-parametric bootstrap resampling (5,000 iterations).

## 4 Discussion

This study provides the first comprehensive longitudinal analysis of performance development in PRO and ELITE HYROX competitions across the first seven competitive seasons. Analyses of the empirical cumulative distribution functions in PRO athletes revealed clear performance improvements from season 4 onward in both sexes, characterized by a distribution-wide shift that was most pronounced at the lower and central percentiles, indicating performance improvements across the competitive spectrum. While performance gains were observed in most exercise stations, the largest reductions in time occurred in the running segments. Nevertheless, running remained the most dominant discipline, consistently accounting for approximately 50% of total race time across seasons. In contrast, exercise stations such as the Ski-Erg, Rowing, and Burpee Broad-Jump showed no or only small performance improvements. Beyond absolute and relative performance trends, the discipline-specific rank reshuffling also showed that Running ranks exhibited the lowest discrepancy to overall ranking, while several non-running exercise stations (e.g., Sled-Push, Sled-Pull and Wallballs) exhibited higher reshuffling values. Thus, Running may primarily act as a discipline to stabilize ranks across the field. Nevertheless, quantile regression confirmed positive associations between discipline times and overall times across all analyzed quantiles, with larger coefficients at higher quantiles, indicating a larger absolute time loss among slower performers in all exercise stations. In ELITE competitions, performance improvements were likewise evident at both the overall and Top-5 levels, accompanied by a notable reduction in performance variability, indicating increasing performance convergence at the highest competitive level.

The percentile-based analyses indicate that performance improvements in PRO competitions from the fourth season onward were not limited to the upper tail of the distribution. Instead, the most pronounced season-to-season changes occurred at the lower percentiles, suggesting a distribution-wide shift in performance rather than isolated improvements among top finishers. Similar patterns have been identified in other endurance sports, such as marathon running, where gradual improvements in race results were observed in the years following the introduction of a female competition category at the Boston marathon in 1972 (Knechtle et al., 2020). Importantly, however, the same study demonstrated that large between-season differences in participation may have a substantial influence on specific segments of the performance distribution, particularly at lower percentiles (Knechtle et al., 2020). Therefore, the apparent decline in PRO performances from season 1 to season 3 is likely attributed to the notion that the large increases in participation of both sexes in the first few seasons lead to a higher heterogeneity in performance levels.

When focusing on the Top 100, a continuous improvement in performance was observed in both male and female PRO competitions from the first season onward. This trend was temporarily disrupted in season 3, which coincided with the global COVID-19 pandemic and a substantially reduced competition calendar, resulting in a markedly lower number of available performances (Fernández-Navarrete et al., 2025). Consequently, the reduced number of performances observed in this season is likely attributable to limited participation, altered competition density, and disrupted training cycles rather than a true performance decline. From Season 4 onwards, the rapid increase in participation was accompanied by progressively faster Top 100 performances. Similar developments have been reported in large-scale 10-km road races in the United States, where improvements in top finishing times coincided with increasing participation and a narrowing performance gap at the front of the field (Cushman et al., 2014). A comparable pattern was evident in the ELITE HYROX competitions, where faster overall and Top 5 performances across seasons were accompanied by a clear reduction in performance variability, indicating increasing competitive density at the highest performance level. Therefore, it can be expected that with holistic long-term development and sport-specific training (Smith, 2003), peak performance of professional HYROX athletes will further improve in the upcoming years.

The discipline-specific analysis of male and female PRO results indicated that the longitudinal performance improvements were not uniformly distributed across exercise stations. In both male and female athletes, the largest absolute gains occurred in Running, even though its relative contribution to the total finishing time remained largely stable at approximately 50% across seasons. Taken together with the lowest discipline-specific rank discrepancy relative to overall ranking, our findings indicate that running is the largest contributor to total race time and that running times were most closely aligned with overall finishing position. Notably, however, when each discipline’s mechanical contribution to the total was removed, running showed only intermediate independent explanatory value. The observed improvements in running may plausibly reflect gains in endurance-related qualities such as V̇O_2_max, lactate thresholds, economy, and resilience (Joyner and Coyle, 2008; Jones, 2024). However, as the present analysis is based on competition times rather than physiological measurements, this remains speculative. Nevertheless, this interpretation is consistent with HYROX-specific physiological data from a simulated competition, where (i) Running accounted for the largest proportion of total competition time, and (ii) a higher V̇O_2_max and higher endurance training volume were associated with a superior overall performance (Brandt et al., 2025). Thus, from a training perspective, these findings emphasize the central role of endurance-orientated training with an increased endurance training volume (Muniz-Pumares et al., 2025), and the inclusions of training blocks of complementary high-intensity interval training sessions (Laursen and Jenkins, 2002; Buchheit and Laursen, 2013a, b) to elicit the desired metabolic stimuli to enhance V̇O_2_max (Lundby et al., 2017) and mimic the high-intensity profile of the most exercise stations in the HYROX competition (Fernández et al., 2015; Tibana et al., 2018; Carreker and Grosicki, 2020; Davids, 2025).

Interestingly, however, other exercises requiring a high aerobic performance level, such as Rowing (Rappelt et al., 2025) or Ski-Erg (Carlsson et al., 2014), exhibited only minor absolute improvements across seasons, potentially reflecting comparatively stronger technical constraints, pacing strategies, and/or earlier ceiling effects. Adversely, strength-related tasks such as Sled-Pull, Farmer’s Carry, Sandbag Lunges, and Wallballs showed larger absolute improvements, but also greater discipline-specific rank discrepancies relative to overall ranking. Importantly, even though the quantile regression analyses demonstrated a systematic increase in coefficients from lower to higher quantiles across all exercise stations, indicating that slower performers show disproportional greater sensitivity of total race time to discipline performance, the strongest quantile-dependent effects were observed in strength-based tasks. This finding suggests that insufficient performance in these non-running exercise stations can substantially amplify overall competition time, particularly among slower athletes. From a training perspective, it highlights the necessity of achieving a sufficient strength capacity to avoid excessive time loss and to perform at a competitive level during the loaded exercises. Additionally, to maintain a high running performance throughout the competition, it seems reasonable to improve core strength, as trunk and upper body fatigue has been shown to detrimentally affect running economy (Drum et al., 2019). However, once a sufficient strength level is attained, further gains in maximal strength are unlikely to translate proportionally into performance improvements, as these gains could be accompanied by increases in body mass, which may negatively impact running performance (Brandt et al., 2025).

Some limitations of this study need to be addressed. First, the data were extracted from a large, publicly accessible database that partially relies on self-reported athlete information (e.g., names and demographics), which may introduce erroneous entries. However, as longitudinal analyses of individual trajectories were restricted to ELITE athletes, particular care was taken to manually harmonize athlete identifiers within this subgroup (e.g., correcting obvious spelling errors or nicknames) to ensure consistency across seasons. As supported by sensitivity analyses, potential errors in self-reported athlete information in PRO results did not relevantly influence our analyses and may thus be considered negligible. Second, while qualification criteria apply for World Championship and ELITE competitions, participation in PRO events does not require meeting predefined performance standards, allowing athletes of heterogeneous performance levels to compete. Consequently, not all PRO participants represent a uniformly high-performance population. Therefore, we decided to perform additional analyses of absolute and relative discipline times using the Top 100, to ensure a high-performance standard. Third, a subset of performances was affected by missing split times, time bonuses (e.g., due to equipment shortages or malfunctions), or penalties (e.g., for incorrectly executed exercises). Thus, to ensure data integrity and comparability across seasons, all affected results were excluded from further analyses, resulting in a reduction of approximately 6% of male and 7.5% of female performances. However, the large remaining dataset and the consistency of results across analytical approaches suggest that this exclusion did not meaningfully influence the study’s conclusions. Finally, although the sequence of and exercise stations themselves are standardized across HYROX competitions and the overall running distance is fixed at 8 km, the exact distance of individual running segments may have varied between events due to differences in the Rox Zone configuration. Consequently, detailed analyses of pacing strategies during the eight running segments were not feasible in the present dataset. Consequently, the observed improvements in running performance may partly reflect changes in pacing strategies rather than physiological adaptations alone, which cannot be disentangled from the available competition data. Notably, however, running segment distances were standardized beginning with the 2024/2025 season, which will enable more detailed analyses of running pacing and modeling in future studies.

Within its first seven seasons since inception, HYROX has rapidly evolved from a novel high-intensity fitness race into a highly competitive, endurance-dominated sport. In this time span, performance improvements occurred not only at elite level but across the full competitive field, with an increasing convergence among top performers indicating a growing professionalization. Although HYROX integrates both endurance- and strength-based tasks, the present findings indicate that overall performance is most strongly associated with running performance. From an applied training perspective, these trends suggest that HYROX athletes should train in a sport-specific manner, with an approach combining endurance sessions, while specifically targeting a repeated strength ability. As participation and competitive density continue to increase, further gains are likely to depend less on general fitness and more on systematic, specialized long-term development. Future research should therefore focus on individual training trajectories, pacing strategies, and physiological profiling to better support performance optimization in this emerging sport.

## Data Availability

Publicly available datasets were analyzed in this study. This data can be found here: https://results.hyrox.com/.
