# Supplementary material for: Longitudinal performance development in PRO and ELITE HYROX competitions across the first seven competitive seasons
Source: Front Physiol. 2026 Jul 8;17:1847569. doi: 10.3389/fphys.2026.1847569 (PMC13409289; doi:10.3389/fphys.2026.1847569)
Supplement: Supplementary file 1 [file DataSheet1.pdf]

Supplementary Material

1 Supplement 1 – Steb-by-step documentation of data extraction pipe-line

Step 1: Listing all competition events:

```
import requests
from bs4 import BeautifulSoup
from time import sleep
from pathlib import Path
import csv

HEADERS = {"User-Agent": "Mozilla/5.0"}
SEASONS = list(range(1, 8+1))  # season-1 ... season-8
PAUSE_S = 7.5                   # höfliche Wartezeit zwischen Requests

def get_soup(base_url: str, params: dict | None = None) -> BeautifulSoup:
    r = requests.get(base_url, params=params, headers=HEADERS, timeout=30)
    r.raise_for_status()
    return BeautifulSoup(r.text, "html.parser")

def extract_options(select_tag):
    """Return list of (value, label) for a <select>, skipping empty/%
placeholders."""
    out = []
    if not select_tag:
        return out
    for opt in select_tag.find_all("option"):
        val = (opt.get("value") or "").strip()
        lab = opt.get_text(strip=True)
        if val and val != "%":
            out.append((val, lab))
    return out

all_rows = []  # will store dicts: season, event_group_id, event_group_name,
event_id, event_name

for season in SEASONS:
    base_url = f"https://results.hyrox.com/season-{season}/"
    print(f"\n=== Season {season}: {base_url} ===")
    try:
        soup0 = get_soup(base_url)
    except Exception as e:
        print(f"[WARN] Season {season} übersprungen (Fehler beim Laden): {e}")
        continue

    group_select = soup0.find("select", attrs={"name": "event_main_group"})
    group_opts = extract_options(group_select)

    if not group_opts:
        print(f"[WARN] Season {season}: keine 'event_main_group'-Optionen
```

```
gefunden.")
        continue

    print(f"  Gefundene Gruppen: {len(group_opts)}")

    for group_val, group_label in group_opts:
        # Seite mit gesetzter Gruppe laden, damit 'event' befüllt wird
        try:
            soup_g = get_soup(base_url, params={"event_main_group": group_val})
        except Exception as e:
            print(f"  [WARN] Gruppe '{group_label}' (val={group_val})
übersprungen: {e}")
            continue

        event_select = soup_g.find("select", attrs={"name": "event"})
        event_opts = extract_options(event_select)

        print(f"  - {group_label}: {len(event_opts)} Events")
        for ev_id, ev_name in event_opts:
            all_rows.append({
                "season": season,
                "event_group_id": group_val,
                "event_group_name": group_label,
                "event_id": ev_id,
                "event_name": ev_name,
            })
        sleep(PAUSE_S)

# Ausgabe: kurz zusammenfassen
print(f"\nGesamt gesammelt: {len(all_rows)} Events über {len(SEASONS)} Seasons.")

# OPTIONAL: als CSV speichern
out_path = Path("hyrox_event_groups_and_events_seasons_1_to_8.csv")
with out_path.open("w", newline="", encoding="utf-8") as f:
    writer = csv.DictWriter(f, fieldnames=[
        "season", "event_group_id", "event_group_name", "event_id", "event_name"
    ])
    writer.writeheader()
    writer.writerows(all_rows)

print(f"CSV gespeichert: {out_path.resolve()}")
```

Then, the list of all results was filtered to contain only PRO and ELITE single competitions (via the event_name label).

Step 2: The raw html code for the overview pages of each competition (i.e., the pages that contained the list of results with links to every athlete's performance during the given competition) was extracted and saved as .csv:

```
import csv
import re
import time
import random
```

```
from pathlib import Path
from urllib.parse import urlencode

import requests

# === CONFIG ===
CSV_PATH = "Pro_competitions_1_8.csv"  # adjust if needed
OUT_DIR = Path("hyrox_html_by_event")
OUT_DIR.mkdir(exist_ok=True)

HEADERS = {"User-Agent": "Mozilla/5.0"}
MAX_PAGES = 10
SLEEP_SECONDS = 7.5  # be polite to the server

# fixed query parts you'd been using
COMMON_PARAMS = {
    "num_results": "100",
    "pid": "list",
    "pidp": "ranking_nav",
    "ranking": "time_finish_netto",
    "search[sex]": "M",
    "search[age_class]": "%",    # all
    "search[nation]": "%",       # all
}

def sanitize_filename(s: str) -> str:
    s = re.sub(r"[^\w\-. ]+", "_", s, flags=re.UNICODE)
    s = re.sub(r"_+", "_", s).strip("_")
    return s[:200]  # avoid absurdly long names

def page_has_no_results(html: str) -> bool:
    # simple heuristics; extend if needed
    needles = ["Keine Ergebnisse", "No results"]
    return any(n in html for n in needles)

def fetch_event_pages_to_txt(season: int, event_group_id: str, event_name: str,
event_id: str):
    base_url = f"https://results.hyrox.com/season-{season}/"
    # output name: season_eventgroupid_eventname.txt
    out_name = f"{season}_{event_group_id}_{event_name}"
    out_file = OUT_DIR / f"{sanitize_filename(out_name)}.txt"

    wrote_any = False
    with out_file.open("w", encoding="utf-8") as f_out:
        for p in range(1, MAX_PAGES + 1):
            params = COMMON_PARAMS.copy()
            params["page"] = str(p)
            params["event"] = event_id

            # Build URL with params safely
            url = base_url + "?" + urlencode(params, doseq=True)
            r = requests.get(url, headers=HEADERS, timeout=30)
            if r.status_code != 200:
                print(f"[{season} | {event_name}] Page {p}: HTTP {r.status_code}
— stop.")
                break

            html = r.text
            if page_has_no_results(html):
```

```
                print(f"[{season} | {event_name}] Page {p}: no results — stop.")
                break

            f_out.write(f"\n\n<!-- ========== START OF PAGE {p} ({url})
========== -->\n\n")
            f_out.write(html)
            f_out.write(f"\n\n<!-- ========== END OF PAGE {p} ========== --
>\n\n")
            print(f"[{season} | {event_name}] appended page {p} →
{out_file.name}")
            wrote_any = True

            time.sleep(random.uniform(5.0, 7.0))


    if not wrote_any:
        out_file.unlink(missing_ok=True)  # remove empty file
        print(f"[{season} | {event_name}] no content; removed empty file.")
    else:
        print(f"[{season} | {event_name}] DONE → {out_file}")

def main():
    # CSV must have: season, event_group_id, event_name, event_id
    with open(CSV_PATH, "r", encoding="cp1252", newline="") as f:
        reader = csv.DictReader(f, delimiter=";")   # <---- add delimiter
        missing_cols = {"season", "event_group_id", "event_name", "event_id"} -
set(reader.fieldnames or [])
        if missing_cols:
            raise ValueError(f"CSV is missing required columns:
{sorted(missing_cols)}")

        for row in reader:
            try:
                season = int(row["season"])
            except Exception:
                print(f"[SKIP] Invalid season value: {row.get('season')!r}")
                continue

            event_group_id = str(row["event_group_id"]).strip()
            event_name = str(row["event_name"]).strip()
            event_id = str(row["event_id"]).strip()

            if not event_id:
                print(f"[SKIP] Missing event_id for row: {row}")
                continue

            fetch_event_pages_to_txt(season, event_group_id, event_name,
event_id)

if __name__ == "__main__":
    main()
```

Step 3: All individual result links from the .html-code were extracted and listed in a .csv-file (we used R for this step):

```r
# Read the file
html <- paste(readLines("1_2018 Essen_HYROX PRO.txt", encoding = "UTF-8"), colla
pse = "\n")

# Extract all links + names
pattern <- '<a href="\\?content[^"]*">(.*?)</a>'
matches <- regmatches(html, gregexpr(pattern, html))

# Unlist the matches
matches <- unlist(matches)
names <- gsub('^<a href="[^"]*">(.*)</a>$', '\\1', matches)

matches <- regmatches(html, gregexpr('href="\\?content[^"]*"', html))

# Clean up (remove href=" and closing ")
links <- gsub('href="|"$', "", unlist(matches))

# Replace &amp and &; with &
links <- gsub("&amp", "&", links)
links <- gsub("&;", "&", links)
links <- links[1:length(names)]

base_url <- "https://results.hyrox.com/season-1/"

links <- paste0(base_url, links)

results <- data.frame(
  name = names,
  link = links,
  stringsAsFactors = FALSE
)

write.csv(
  results,
  file = "Season_01_Essen.csv",
  row.names = FALSE
)
```

Step 4: These .csv-files (containing all links by name) were used to open every individual link to all individual performances per competition and extract all individual performance data into a single .xlsx-file with multiple sheets:

```python
from bs4 import BeautifulSoup
import pandas as pd
import requests, io, re
from pathlib import Path
from urllib.parse import urlparse, parse_qs, unquote
import random
```

```
import time

def get_event_code(url: str) -> str:
    """Event-Code aus der URL extrahieren (nach 'event=' bis vor dem nächsten
'&')."""
    try:
        parsed = urlparse(url)
        qs = parse_qs(parsed.query)
        if "event" in qs and qs["event"]:
            code = unquote(qs["event"][0])
        else:
            # Fallback: Regex
            m = re.search(r'(?:[?&])event=([^&]+)', url)
            code = unquote(m.group(1)) if m else "UnknownEvent"
    except Exception:
        code = "UnknownEvent"
    # Für Dateinamen säubern und etwas kürzen
    code = "".join(c if (c.isalnum() or c in " -_") else "_" for c in
code).strip()[:80]
    return code or "UnknownEvent"

# CSV mit Spalten "name" und "link" einlesen
df_links = pd.read_csv("males_season_1-8.csv", encoding='latin1')

# Ausgabeordner
out_dir = Path("athlete_excels_S1_8")
out_dir.mkdir(exist_ok=True)

for idx, row in df_links.iterrows():
    name = str(row["name"]).strip()
    url = str(row["link"]).strip()

    if not url or not name:
        continue

    # Event-Code aus dem Link holen
    event_code = get_event_code(url)

    # Dateiname vorbereiten (illegale Zeichen entfernen, Länge begrenzen)
    safe_name = "".join(c if c.isalnum() or c in " _-" else "_" for c in
name)[:100]
    out_path = out_dir / f"{event_code}_{safe_name}.xlsx"

    print(f"[{idx+1}/{len(df_links)}] Hole Daten für {name} …")

    try:
        req = requests.get(url, timeout=30)
        req.raise_for_status()
        soup = BeautifulSoup(req.text, "html.parser")

        h3s = soup.find_all("h3")

        with pd.ExcelWriter(out_path, engine="openpyxl") as writer:
            for i in range(7):  # Tabellen 0..6
                if i >= len(h3s):
                    continue
                header = h3s[i].get_text(strip=True)
```

```
                table = h3s[i].find_next("table")

                if table is None:
                    continue

                dfs = pd.read_html(io.StringIO(str(table)))
                if not dfs:
                    continue

                df = dfs[0]
                # Sheetname säubern
                safe_header = header.replace("/", "-").replace("\\", "-
").replace(":", "-")
                if not safe_header:
                    safe_header = f"Table_{i}"
                sheet_name = (safe_header[:28] + f"_{i}") if len(safe_header) >
31 else f"{safe_header}_{i}"

                df.to_excel(writer, index=False, sheet_name=sheet_name)


        print(f"   → gespeichert: {out_path}")

    except Exception as e:
        print(f"   Fehler bei {name}: {e}")

    time.sleep(random.uniform(7.5, 12.5))
```

Step 5: All individual files for males and females were then condensed into a single table (we used R for this step):

```r
library(readxl)
library(stringr)
library(dplyr)
library(tibble)
library(tools)

.normalize <- function(x) gsub("[^a-z]", "", tolower(x))

get_sheet_index <- function(path, targets = c("workout summary", "race rep
lay")) {
  sheets <- excel_sheets(path)
  norm   <- .normalize(sheets)
  # normalize targets similarly
  tnorm  <- .normalize(targets)

  # find first matching target by prefix
  for (t in tnorm) {
    idx <- which(startsWith(norm, t))
    if (length(idx) > 0) return(idx[1])
  }
```

```
  NA_integer_
}
df <- c()

files <- list.files(pattern = "*.xls")


for (i in 1:length(files)){

  if (length(excel_sheets(files[i])) < 5) next

  df_1 <- read_xlsx(files[i], sheet = 1)
  df_2 <- read_xlsx(files[i], sheet = 2)
  df_3 <- read_xlsx(files[i], sheet = 3)
  df_4 <- read_xlsx(files[i], sheet = 4)

  if (is.na(get_sheet_index(files[i], targets = c("workout summary")))) ne
xt

  df_6 <- read_xlsx(files[i], sheet = get_sheet_index(files[i], targets =
c("workout summary")))

  if (is.na(get_sheet_index(files[i], targets = c("race replay")))) next
  df_7 <- read_xlsx(files[i], sheet = get_sheet_index(files[i], targets =
c("race replay")))

  name <- as.character(df_1[which(df_1[, 1] == "Name"), 2])
  ID <- as.character(df_1[which(df_1[, 1] == "Bib Number"), 2])
  age_group <- as.character(df_1[which(df_1[, 1] == "Age Group"), 2])
  nation <- as.character(df_1[which(df_1[, 1] == "Nat"), 2])

  division <- as.character(df_2[which(df_2[, 1] == "Division"), 2])
  race <- as.character(df_2[which(df_2[, 1] == "Race"), 2])
  race_ID <- str_match(file_path_sans_ext(basename(files[i])), "^([A-Za-z]
+_[A-Za-z0-9]+)_(.+?)(?:_\\s*([A-Za-z]{3})_?)?$")[2]

  rank <- as.character(df_4[which(df_4[, 1] == "Rank (M/W)"), 2])
  rank_age_group <- as.character(df_4[which(df_4[, 1] == "Rank (AG)"), 2])
  overall_time <- as.character(df_4[which(df_4[, 1] == "Overall Time"), 2]
)

  bonus <- as.character(df_3[which(df_3[, 1] == "*Bonus"), 2])
  penalty <- as.character(df_3[which(df_3[, 1] == "*Penalty"), 2])
  disqual <- as.character(df_3[which(df_3[, 1] == "Disqual Reason"), 2])
  info <- as.character(df_3[which(df_3[, 1] == "Info"), 2])
```

```
  ID_Race_Div_Rank_Penalty <- c(name, ID, age_group, nation, division, rac
e, race_ID, rank, rank_age_group, overall_time, bonus, penalty, disqual, i
nfo)

  Running <- c(as.character(df_6[which(df_6$Split == "Running 1"), 2]),
               as.character(df_6[which(df_6$Split == "Running 2"), 2]),
               as.character(df_6[which(df_6$Split == "Running 3"), 2]),
               as.character(df_6[which(df_6$Split == "Running 4"), 2]),
               as.character(df_6[which(df_6$Split == "Running 5"), 2]),
               as.character(df_6[which(df_6$Split == "Running 6"), 2]),
               as.character(df_6[which(df_6$Split == "Running 7"), 2]),
               as.character(df_6[which(df_6$Split == "Running 8"), 2]),
               as.character(df_6[which(df_6$Split == "Run Total"), 2]))

  Exercises <- c(as.character(df_6[which(df_6$Split == "1000m SkiErg"), 2]
),
                 as.character(df_6[which(df_6$Split == "50m Sled Push"), 2
]),
                 as.character(df_6[which(df_6$Split == "50m Sled Pull"), 2
]),
                 as.character(df_6[which(df_6$Split == "80m Burpee Broad J
ump"), 2]),
                 as.character(df_6[which(df_6$Split == "1000m Row"), 2]),
                 as.character(df_6[which(df_6$Split == "200m Farmers Carry
"), 2]),
                 as.character(df_6[which(df_6$Split == "100m Sandbag Lunge
s"), 2]),
                 as.character(df_6[which(df_6$Split == "Wall Balls"), 2]),
                 as.character(df_6[which(df_6$Split == "Roxzone Time"), 2]
))

  if (length(c(which(df_7$Split == "Rox In"))) == 7){

    Roxtime <- as.numeric(as.difftime(unlist(as.vector(df_7[c(which(df_7$S
plit == "Rox In") + 1), ncol(df_7)])), format = "%M:%S", units = "secs"))
+
      as.numeric(as.difftime(unlist(as.vector(df_7[c(which(df_7$Split == "
Rox Out")), ncol(df_7)])), format = "%M:%S", units = "secs"))

    Roxtime_total <- sum(Roxtime)

  } else {
    Roxtime <- rep(NA, 7)

    if (length(c(which(df_6$Split == "Roxzone Time"))) == 1){
      Roxtime_total <- as.numeric(as.difftime(df_6$Time[(which(df_6$Split
```

```
== "Roxzone Time"))], format = "%H:%M:%S", units = "secs"))
    } else {
      Roxtime_total <- NA
    }
  }


  results <- c(ID_Race_Div_Rank_Penalty, Running, Exercises, Roxtime, Roxt
ime_total)

  df <- rbind(df, results)

  print(i/length(files)) * 100
}

df_all <- as.data.frame(df)

colnames(df_all) <- c("name", "ID", "age_group", "nation", "dividion", "ra
ce", "race_ID", "rank", "rank_age_group",
                       "overall_time", "bonus", "penalty", "disqual", "info
", "Running 1", "Running 2", "Running 3", "Running 4", "Running 5",
                       "Running 6", "Running 7", "Running 8", "Running_tota
l", "1000m_SkiErg", "50m_Sled_Push", "50m_Sled_Pull", "80m_Burpee_Broad_Ju
mp",
                       "1000m_Row", "200m_Farmers Carry", "100m_Sandbag_Lun
ges", "Wall_Balls", "Roxzone_Time", "R1", "R2", "R3", "R4", "R5", "R6", "R
7", "Roxtime_total")

rownames(df_all) <- NULL


writexl::write_xlsx(df_all, "overview_males.xlsx")
```

Final remarks:

The retrieval ceiling (up to 10 pages of 100 results) was never a limiting factor for any included PRO event in either sex, so no slower finishers were truncated. Parts of Season 8 were retrieved but excluded from all analyses, as it had only just commenced at the extraction date and was incomplete.

Supplementary Table S2. Mean percentile-specific differences in finishing time with 95% bootstrap confidence intervals. Upper triangle displays results for women (grey), lower triangle for men (transparent). Negative values indicate faster performance in the later season. Values printed in bold indicate $p < 0.05$ after global Holm–Bonferroni correction.

	Season 1	Season 2	Season 3	Season 4	Season 5	Season 6	Season 7
Season 1		P25: -3 [-162; +143]<br>P50: +72 [-130; +265]<br>P75: +230 [-36; +446]<br>P90: +346 [+0; +813]	P25: +172 [-105; +558]<br>P50: +405 [-158; +928]<br>P75: +566 [+86; +1552]<br>P90: +1251 [+169; +1541]	P25: -81 [-191; +31]<br>P50: -98 [-257; +80]<br>P75: -146 [-289; +36]<br>P90: -61 [-298; +259]	P25: -173 [-270; -60]<br>P50: -204 [-354; -32]<br>P75: -214 [-359; -67]<br>P90: -17 [-266; +225]	P25: -191 [-287; -84]<br>P50: -181 [-337; -21]<br>P75: -138 [-269; +15]<br>P90: +140 [-117; +385]	P25: -220 [-311; -118]<br>P50: -144 [-299; +27]<br>P75: -56 [-188; +83]<br>P90: +231 [+5; +446]
Season 2	**P25: +171 [+58; +301]**<br>**P50: +245 [+117; +345]**<br>P75: +203 [+39; +387]<br>P90: +124 [-94; +440]		P25: +175 [-110; +574]<br>P50: +333 [-196; +826]<br>P75: +336 [-137; +1334]<br>P90: +905 [-232; +1194]	P25: -78 [-198; +54]<br>P50: -170 [-285; -39]<br>P75: -376 [-563; -118]<br>P90: -407 [-851; -76]	P25: -170 [-285; -46]<br>**P50: -276 [-376; -152]**<br>**P75: -445 [-634; -208]**<br>P90: -363 [-817; -109]	P25: -188 [-305; -63]<br>**P50: -254 [-354; -134]**<br>**P75: -369 [-549; -128]**<br>P90: -206 [-656; +65]	**P25: -217 [-335; -103]**<br>**P50: -217 [-312; -100]**<br>P75: -286 [-461; -60]<br>P90: -115 [-570; +119]
Season 3	**P25: +364 [+191; +535]**<br>**P50: +449 [+151; +612]**<br>P75: +441 [+105; +995]<br>P90: +621 [+211; +872]	P25: +193 [+21; +357]<br>P50: +204 [-75; +366]<br>P75: +238 [-113; +770]<br>P90: +497 [+73; +643]		P25: -253 [-584; +7]<br>P50: -503 [-984; +35]<br>P75: -712 [-1701; -228]<br>P90: -1312 [-1507; -174]	P25: -345 [-674; -97]<br>P50: -609 [-1088; -100]<br>**P75: -780 [-1778; -325]**<br>P90: -1267 [-1490; -212]	P25: -363 [-703; -105]<br>P75: -704 [-1707; -245]<br>P50: -586 [-1071; -38]<br>P90: -1110 [-1324; -27]	**P25: -392 [-725; -138]**<br>P75: -622 [-1618; -182]<br>P50: -550 [-998; -18]<br>P90: -1019 [-1214; +27]
Season 4	P25: -141 [-224; -40]<br>**P50: -134 [-242; -61]**<br>P75: -187 [-325; -43]<br>P90: -260 [-482; -7]	**P25: -312 [-406; -222]**<br>**P50: -380 [-473; -304]**<br>**P75: -390 [-527; -262]**<br>**P90: -384 [-614; -251]**	**P25: -505 [-654; -345]**<br>**P50: -584 [-745; -300]**<br>**P75: -628 [-1148; -297]**<br>**P90: -880 [-1040; -473]**		P25: -92 [-161; -12]<br>P50: -106 [-184; -32]<br>P75: -68 [-203; +24]<br>P90: +44 [-221; +185]	P25: -110 [-174; -31]<br>P50: -83 [-160; -17]<br>P75: +8 [-114; +109]<br>P90: +202 [-65; +358]	**P25: -139 [-197; -63]**<br>P50: -46 [-118; +24]<br>P75: +90 [-27; +165]<br>P90: +293 [+48; +405]
Season 5	**P25: -298 [-373; -202]**<br>**P50: -248 [-360; -181]**<br>**P75: -345 [-480; -217]**<br>P90: -354 [-563; -104]	**P25: -469 [-557; -386]**<br>**P50: -493 [-587; -430]**<br>**P75: -548 [-685; -441]**<br>**P90: -478 [-704; -363]**	**P25: -662 [-797; -500]**<br>**P50: -697 [-862; -416]**<br>**P75: -785 [-1296; -475]**<br>**P90: -975 [-1121; -608]**	**P25: -157 [-205; -107]**<br>**P50: -114 [-169; -72]**<br>**P75: -158 [-259; -85]**<br>P90: -94 [-212; +27]		P25: -18 [-71; +36]<br>P50: +22 [-35; +72]<br>P75: +76 [+0; +170]<br>P90: +157 [+2; +326]	P25: -47 [-90; -1]<br>P50: +59 [+7; +109]<br>**P75: +158 [+79; +232]**<br>**P90: +248 [+126; +377]**
Season 6	**P25: -253 [-326; -159]**<br>**P50: -179 [-286; -116]**<br>**P75: -201 [-323; -80]**<br>P90: -221 [-438; +11]	**P25: -424 [-510; -343]**<br>**P50: -424 [-517; -360]**<br>**P75: -404 [-536; -301]**<br>**P90: -345 [-572; -242]**	**P25: -617 [-752; -454]**<br>**P50: -628 [-783; -334]**<br>**P75: -642 [-1146; -321]**<br>**P90: -841 [-994; -449]**	**P25: -112 [-158; -69]**<br>P50: -44 [-96; -5]<br>P75: -14 [-107; +54]<br>P90: +39 [-84; +144]	P25: +45 [+6; +77]<br>**P50: +69 [+33; +108]**<br>**P75: +143 [+92; +204]**<br>P90: +133 [+31; +211]		P25: -29 [-70; +11]<br>P50: +36 [-5; +83]<br>P75: +82 [-1; +137]<br>P90: +91 [-47; +216]
Season 7	**P25: -317 [-387; -224]**<br>**P50: -235 [-343; -173]**<br>**P75: -226 [-346; -107]**<br>P90: -181 [-385; +48]	**P25: -488 [-574; -409]**<br>**P50: -480 [-571; -420]**<br>**P75: -429 [-550; -329]**<br>**P90: -305 [-526; -205]**	**P25: -681 [-813; -518]**<br>**P50: -684 [-839; -390]**<br>**P75: -667 [-1164; -353]**<br>**P90: -802 [-941; -438]**	**P25: -176 [-221; -135]**<br>**P50: -100 [-148; -63]**<br>P75: -39 [-128; +25]<br>P90: +79 [-28; +180]	P25: -19 [-56; +9]<br>P50: +13 [-18; +49]<br>**P75: +119 [+69; +179]**<br>**P90: +173 [+79; +248]**	**P25: -64 [-91; -39]**<br>**P50: -56 [-86; -27]**<br>P75: -25 [-64; +17]<br>P90: +40 [-29; +114]	

Supplementary Table S3. Mean percentile-specific differences in finishing time (seconds) with 95% bootstrap confidence intervals. Upper triangle displays results for women (grey), lower triangle for men (transparent). Negative values indicate faster performance in the later season. Values printed in bold indicate $p < 0.05$ after global Holm–Bonferroni correction. Confidence intervals and *p*-values are based on one-performance-per-athlete resampling (fastest time retained per athlete per season) to account for repeated performances.

	Season 1	Season 2	Season 3	Season 4	Season 5	Season 6	Season 7
Season 1		P25: +65 [-85; +276]<br>P50: +169 [-27; +392]<br>P75: +373 [+72; +577]<br>P90: +503 [+116; +994]	P25: +162 [-111; +526]<br>P50: +276 [-157; +871]<br>P75: +555 [+62; +1542]<br>P90: +1270 [+50; +1544]	P25: +5 [-113; +155]<br>P50: -32 [-220; +177]<br>P75: -31 [-234; +166]<br>P90: +202 [-160; +427]	P25: -127 [-228; +16]<br>P50: -107 [-289; +89]<br>P75: -124 [-289; +64]<br>P90: +79 [-211; +301]	P25: -142 [-234; -3]<br>P50: -91 [-261; +95]<br>P75: -33 [-183; +153]<br>P90: +327 [+54; +602]	P25: -115 [-210; +27]<br>P50: +12 [-154; +199]<br>P75: +88 [-61; +273]<br>P90: +413 [+164; +625]
Season 2	**P25: +289 [+191; +401]**<br>**P50: +302 [+149; +415]**<br>P75: +275 [+81; +464]<br>P90: +147 [-92; +512]		P25: +97 [-206; +421]<br>P50: +107 [-327; +643]<br>P75: +182 [-307; +1159]<br>P90: +767 [-521; +1101]	P25: -60 [-234; +86]<br>P50: -201 [-383; -62]<br>P75: -405 [-588; -112]<br>P90: -301 [-822; -10]	P25: -192 [-345; -53]<br>**P50: -276 [-434; -153]**<br>**P75: -497 [-640; -207]**<br>P90: -424 [-884; -114]	P25: -207 [-350; -67]<br>**P50: -260 [-423; -142]**<br>**P75: -406 [-531; -115]**<br>P90: -175 [-618; +160]	P25: -181 [-332; -43]<br>P50: -158 [-331; -41]<br>P75: -285 [-400; -8]<br>P90: -89 [-514; +199]
Season 3	**P25: +372 [+200; +555]**<br>**P50: +445 [+169; +640]**<br>P75: +359 [+48; +967]<br>P90: +448 [+91; +847]	P25: +83 [-81; +247]<br>P50: +143 [-125; +333]<br>P75: +84 [-220; +655]<br>P90: +301 [-83; +604]		P25: -157 [-474; +103]<br>P50: -308 [-858; +123]<br>P75: -586 [-1592; -80]<br>P90: -1068 [-1379; +115]	P25: -288 [-602; -29]<br>P50: -383 [-919; +34]<br>P75: -678 [-1662; -185]<br>P90: -1191 [-1427; +8]	P25: -304 [-655; -49]<br>P50: -367 [-897; +50]<br>P75: -587 [-1581; -91]<br>P90: -943 [-1164; +253]	P25: -277 [-574; -31]<br>P50: -265 [-776; +147]<br>P75: -466 [-1451; +16]<br>P90: -857 [-1061; +322]
Season 4	P25: -50 [-142; +56]<br>P50: -38 [-168; +33]<br>P75: -95 [-226; +85]<br>P90: -236 [-459; +71]	**P25: -339 [-415; -267]**<br>**P50: -340 [-451; -250]**<br>**P75: -369 [-504; -201]**<br>**P90: -383 [-645; -205]**	**P25: -422 [-585; -265]**<br>**P50: -483 [-695; -225]**<br>**P75: -454 [-1015; -144]**<br>**P90: -684 [-989; -359]**		P25: -131 [-211; -38]<br>P50: -75 [-186; +22]<br>P75: -92 [-224; +69]<br>P90: -123 [-291; +159]	**P25: -147 [-216; -67]**<br>P50: -60 [-153; +27]<br>P75: -1 [-122; +151]<br>P90: +125 [-40; +439]	P25: -120 [-193; -45]<br>P50: +43 [-44; +124]<br>P75: +120 [+9; +271]<br>**P90: +212 [+86; +483]**
Season 5	**P25: -182 [-268; -80]**<br>**P50: -171 [-295; -98]**<br>**P75: -274 [-401; -110]**<br>P90: -296 [-488; +1]	**P25: -472 [-539; -403]**<br>**P50: -473 [-573; -379]**<br>**P75: -549 [-677; -395]**<br>**P90: -443 [-692; -267]**	**P25: -555 [-712; -398]**<br>**P50: -616 [-812; -393]**<br>**P75: -633 [-1192; -335]**<br>**P90: -744 [-1032; -416]**	**P25: -133 [-185; -68]**<br>**P50: -133 [-186; -68]**<br>P75: -180 [-290; -82]<br>P90: -60 [-186; +95]		P25: -15 [-76; +44]<br>P50: +16 [-63; +98]<br>P75: +91 [-4; +194]<br>P90: +248 [+74; +465]	P25: +11 [-53; +61]<br>**P50: +118 [+46; +193]**<br>**P75: +212 [+124; +313]**<br>**P90: +335 [+192; +497]**
Season 6	**P25: -170 [-254; -76]**<br>P50: -105 [-227; -34]<br>P75: -139 [-261; +14]<br>P90: -210 [-383; +86]	**P25: -460 [-526; -398]**<br>**P50: -407 [-506; -319]**<br>**P75: -414 [-539; -266]**<br>**P90: -357 [-590; -202]**	**P25: -543 [-715; -385]**<br>**P50: -549 [-759; -321]**<br>**P75: -498 [-1052; -207]**<br>**P90: -658 [-949; -337]**	**P25: -121 [-167; -66]**<br>P50: -67 [-114; -6]<br>P75: -44 [-144; +37]<br>P90: +26 [-82; +159]	P25: +12 [-39; +54]<br>P50: +67 [+15; +118]<br>**P75: +135 [+50; +211]**<br>P90: +86 [-20; +188]		P25: +26 [-25; +68]<br>P50: +103 [+44; +155]<br>P75: +121 [+44; +200]<br>P90: +86 [-92; +241]
Season 7	**P25: -182 [-266; -93]**<br>**P50: -128 [-251; -68]**<br>P75: -153 [-265; -4]<br>P90: -153 [-326; +134]	**P25: -472 [-538; -411]**<br>**P50: -430 [-526; -348]**<br>**P75: -427 [-548; -281]**<br>**P90: -300 [-547; -150]**	**P25: -555 [-717; -398]**<br>**P50: -572 [-781; -349]**<br>**P75: -512 [-1059; -212]**<br>**P90: -601 [-914; -285]**	**P25: -133 [-178; -83]**<br>**P50: -90 [-134; -36]**<br>P75: -58 [-151; +25]<br>P90: +83 [-23; +215]	P25: +0 [-51; +34]<br>P50: +44 [-4; +84]<br>P75: +121 [+45; +190]<br>P90: +143 [+38; +230]	P25: -12 [-47; +14]<br>P50: -23 [-68; +11]<br>P75: -14 [-69; +43]<br>P90: +57 [-26; +121]	

Supplementary Table S4: Quantile regression coefficients (leave-one-out) for the association between standardized discipline times and total race time excluding the focal discipline in male athletes, stratified by season for selected conditional quantiles (τ). Values represent regression coefficients in seconds per 1 SD increase in discipline time, with 95% confidence intervals in parentheses. Lower τ values correspond to faster performances. The leave-one-out outcome (total time minus the focal discipline) removes the mechanical part-whole contribution and reflects independent predictive value.

Discipline	τ	Season 1	Season 2	Season 3	Season 4	Season 5	Season 6	Season 7
Running	0.10	285 (242 - 327)	331 (294 - 367)	163 (53 - 272)	317 (295 - 339)	284 (266 - 302)	278 (260 - 297)	318 (309 - 326)
	0.25	316 (273 - 359)	376 (340 - 412)	241 (131 - 352)	347 (324 - 369)	354 (338 - 370)	342 (329 - 354)	381 (373 - 389)
	0.50	386 (343 - 429)	390 (354 - 426)	426 (268 - 583)	408 (388 - 428)	401 (384 - 417)	407 (394 - 419)	445 (436 - 453)
	0.75	436 (367 - 504)	425 (354 - 496)	440 (275 - 605)	462 (434 - 490)	461 (438 - 484)	481 (465 - 497)	518 (506 - 530)
Ski-Erg	0.10	452 (385 - 518)	505 (445 - 564)	567 (431 - 703)	393 (352 - 434)	432 (408 - 456)	482 (464 - 501)	508 (496 - 520)
	0.25	535 (485 - 585)	558 (500 - 616)	530 (402 - 658)	456 (425 - 487)	505 (481 - 528)	540 (523 - 557)	586 (574 - 598)
	0.50	613 (552 - 675)	588 (535 - 641)	625 (458 - 791)	502 (461 - 543)	574 (548 - 600)	631 (612 - 650)	672 (659 - 686)
	0.75	673 (587 - 759)	661 (581 - 741)	599 (368 - 829)	606 (558 - 654)	655 (623 - 687)	691 (665 - 717)	757 (738 - 776)
Sled-Push	0.10	378 (289 - 468)	310 (207 - 414)	500 (372 - 629)	280 (241 - 318)	414 (383 - 446)	376 (350 - 402)	347 (325 - 368)
	0.25	421 (353 - 490)	382 (290 - 475)	504 (415 - 594)	300 (259 - 341)	483 (449 - 518)	469 (442 - 495)	491 (474 - 509)
	0.50	550 (478 - 622)	444 (346 - 543)	456 (349 - 563)	335 (290 - 379)	556 (521 - 592)	566 (539 - 593)	585 (567 - 603)
	0.75	584 (477 - 691)	597 (473 - 721)	620 (354 - 885)	373 (305 - 442)	647 (596 - 697)	644 (612 - 676)	662 (641 - 684)
Sled-Pull	0.10	313 (173 - 452)	298 (198 - 398)	256 (-12 - 524)	305 (258 - 352)	333 (296 - 370)	384 (357 - 410)	424 (406 - 442)
	0.25	382 (297 - 467)	365 (292 - 438)	269 (138 - 401)	377 (339 - 416)	432 (398 - 466)	500 (478 - 521)	533 (516 - 550)
	0.50	533 (450 - 615)	384 (298 - 470)	234 (74 - 394)	455 (415 - 495)	596 (563 - 629)	604 (582 - 625)	676 (660 - 692)
	0.75	640 (499 - 780)	565 (480 - 650)	232 (-14 - 478)	606 (545 - 666)	716 (675 - 756)	701 (671 - 730)	826 (803 - 848)
Burpee Broad-Jump	0.10	486 (430 - 542)	505 (427 - 584)	537 (370 - 704)	485 (447 - 523)	492 (469 - 515)	499 (485 - 513)	530 (518 - 541)
	0.25	543 (482 - 604)	543 (493 - 593)	593 (457 - 730)	531 (502 - 560)	534 (512 - 555)	552 (537 - 566)	597 (588 - 607)
	0.50	587 (526 - 648)	570 (516 - 624)	478 (380 - 576)	563 (530 - 597)	597 (571 - 623)	608 (591 - 624)	659 (647 - 671)
	0.75	613 (535 - 692)	588 (511 - 665)	391 (278 - 503)	624 (574 - 674)	686 (655 - 717)	666 (642 - 690)	738 (722 - 754)
Rowing	0.10	497 (448 - 545)	566 (493 - 639)	638 (543 - 733)	534 (482 - 586)	513 (486 - 540)	551 (533 - 570)	574 (562 - 586)
	0.25	568 (516 - 620)	673 (627 - 719)	609 (485 - 734)	650 (599 - 701)	610 (588 - 631)	635 (619 - 651)	672 (661 - 683)
	0.50	628 (580 - 677)	724 (675 - 774)	627 (473 - 781)	733 (691 - 774)	705 (680 - 730)	735 (717 - 753)	777 (766 - 787)

	0.75	751 (663 - 839)	806 (733 - 879)	693 (384 - 1002)	873 (826 - 920)	817 (786 - 847)	834 (811 - 856)	885 (871 - 899)
Farmers Carry	0.10	334 (243 - 424)	509 (432 - 586)	486 (222 - 750)	436 (388 - 485)	495 (465 - 525)	473 (453 - 492)	484 (469 - 499)
	0.25	443 (371 - 515)	530 (473 - 587)	505 (319 - 690)	497 (459 - 535)	561 (534 - 588)	549 (529 - 570)	583 (569 - 598)
	0.50	528 (444 - 613)	547 (498 - 596)	629 (424 - 835)	575 (533 - 616)	637 (605 - 670)	640 (618 - 662)	674 (660 - 689)
	0.75	666 (566 - 766)	608 (520 - 696)	690 (496 - 883)	676 (624 - 728)	696 (662 - 731)	710 (682 - 737)	767 (750 - 784)
Sandbag Lunges	0.10	457 (385 - 530)	488 (383 - 594)	376 (302 - 449)	460 (413 - 507)	475 (445 - 504)	506 (488 - 524)	517 (505 - 529)
	0.25	549 (482 - 616)	569 (514 - 624)	509 (353 - 665)	527 (491 - 563)	569 (542 - 595)	581 (567 - 596)	612 (601 - 623)
	0.50	617 (559 - 674)	661 (607 - 715)	522 (352 - 693)	622 (582 - 662)	656 (626 - 686)	678 (658 - 697)	720 (708 - 733)
	0.75	702 (615 - 789)	775 (696 - 855)	685 (442 - 928)	690 (640 - 741)	730 (692 - 769)	750 (726 - 775)	845 (826 - 864)
Wallballs	0.10	235 (144 - 325)	364 (260 - 469)	492 (299 - 686)	360 (318 - 401)	371 (338 - 405)	386 (363 - 410)	423 (409 - 438)
	0.25	342 (261 - 422)	450 (392 - 507)	518 (395 - 641)	410 (365 - 456)	447 (422 - 473)	477 (454 - 501)	517 (504 - 531)
	0.50	441 (361 - 520)	469 (399 - 540)	512 (384 - 640)	491 (451 - 532)	523 (490 - 557)	574 (550 - 598)	635 (619 - 650)
	0.75	498 (392 - 605)	592 (501 - 682)	580 (311 - 849)	586 (539 - 634)	646 (602 - 691)	675 (645 - 705)	744 (725 - 762)

Supplementary Table S5: Quantile regression coefficients (leave-one-out) for the association between standardized discipline times and total race time excluding the focal discipline in female athletes, stratified by season for selected conditional quantiles (τ). Values represent regression coefficients in seconds per 1 SD increase in discipline time, with 95% confidence intervals in parentheses. Lower τ values correspond to faster performances. The leave-one-out outcome (total time minus the focal discipline) removes the mechanical part-whole contribution and reflects independent predictive value.

Discipline	τ	Season 1	Season 2	Season 3	Season 4	Season 5	Season 6	Season 7
Running	0.10	263 (182 - 344)	323 (258 - 388)	254 (215 - 292)	293 (257 - 329)	266 (239 - 292)	240 (206 - 274)	302 (287 - 318)
	0.25	281 (211 - 351)	391 (339 - 442)	219 (75 - 363)	333 (302 - 363)	306 (284 - 328)	317 (295 - 339)	370 (356 - 384)
	0.50	352 (273 - 431)	438 (361 - 515)	265 (-94 - 623)	355 (321 - 389)	356 (328 - 385)	393 (371 - 416)	452 (438 - 465)
	0.75	427 (342 - 512)	491 (393 - 589)	671 (245 - 1097)	408 (364 - 452)	396 (366 - 427)	489 (462 - 516)	535 (516 - 554)
Ski-Erg	0.10	339 (247 - 431)	337 (208 - 466)	664 (343 - 986)	371 (329 - 412)	446 (401 - 492)	442 (415 - 470)	481 (460 - 502)
	0.25	384 (297 - 471)	495 (398 - 592)	594 (329 - 859)	430 (372 - 487)	516 (468 - 565)	501 (472 - 529)	569 (551 - 587)
	0.50	485 (386 - 583)	611 (503 - 718)	554 (198 - 909)	494 (439 - 549)	557 (522 - 591)	602 (569 - 636)	678 (657 - 699)
	0.75	564 (428 - 701)	770 (646 - 894)	590 (117 - 1063)	566 (493 - 639)	596 (530 - 662)	715 (671 - 760)	785 (758 - 812)
Sled-Push	0.10	189 (62 - 316)	171 (24 - 317)	207 (169 - 244)	219 (164 - 273)	321 (267 - 375)	400 (372 - 427)	269 (249 - 288)
	0.25	255 (84 - 426)	265 (88 - 442)	440 (87 - 792)	311 (240 - 381)	392 (342 - 442)	431 (403 - 458)	378 (351 - 405)
	0.50	422 (290 - 555)	534 (342 - 726)	588 (45 - 1130)	328 (280 - 376)	460 (410 - 509)	536 (503 - 569)	517 (489 - 545)
	0.75	485 (291 - 680)	610 (393 - 828)	397 (-103 - 897)	309 (223 - 396)	549 (484 - 614)	630 (585 - 674)	635 (600 - 669)
Sled-Pull	0.10	246 (37 - 456)	270 (102 - 439)	455 (217 - 692)	277 (230 - 323)	322 (276 - 368)	337 (297 - 377)	394 (366 - 421)
	0.25	228 (114 - 341)	425 (302 - 549)	551 (156 - 946)	377 (310 - 444)	408 (365 - 451)	437 (403 - 470)	514 (489 - 538)
	0.50	384 (241 - 527)	484 (376 - 592)	757 (334 - 1180)	478 (418 - 537)	478 (431 - 526)	551 (519 - 583)	651 (625 - 677)
	0.75	425 (225 - 625)	639 (472 - 806)	656 (206 - 1107)	538 (458 - 617)	599 (544 - 653)	703 (653 - 753)	820 (783 - 857)
Burpee Broad-Jump	0.10	344 (168 - 519)	451 (318 - 584)	641 (39 - 1244)	473 (418 - 528)	457 (420 - 495)	528 (493 - 564)	584 (573 - 595)
	0.25	552 (440 - 663)	582 (462 - 702)	751 (518 - 985)	497 (451 - 542)	517 (484 - 549)	605 (578 - 632)	630 (614 - 646)
	0.50	634 (546 - 723)	665 (577 - 754)	635 (396 - 874)	531 (482 - 581)	547 (514 - 580)	658 (627 - 690)	690 (671 - 708)
	0.75	567 (518 - 617)	751 (661 - 842)	698 (240 - 1155)	585 (541 - 629)	576 (530 - 622)	732 (692 - 771)	763 (740 - 786)
Rowing	0.10	316 (220 - 411)	448 (352 - 544)	540 (241 - 839)	459 (413 - 506)	458 (421 - 495)	484 (460 - 509)	535 (517 - 553)
	0.25	397 (328 - 466)	524 (445 - 603)	675 (400 - 951)	516 (469 - 563)	488 (457 - 519)	581 (555 - 607)	632 (612 - 651)
	0.50	558 (468 - 647)	614 (525 - 704)	779 (578 - 980)	553 (503 - 602)	558 (523 - 592)	688 (661 - 714)	739 (720 - 757)

	0.75	568 (438 - 698)	671 (527 - 815)	808 (449 - 1168)	636 (582 - 691)	636 (595 - 678)	805 (766 - 844)	864 (841 - 887)
Farmers Carry	0.10	355 (221 - 490)	480 (361 - 599)	499 (453 - 544)	427 (360 - 495)	435 (385 - 484)	443 (409 - 478)	468 (444 - 492)
	0.25	456 (350 - 562)	517 (439 - 595)	535 (315 - 755)	483 (422 - 544)	466 (424 - 508)	538 (512 - 563)	565 (542 - 587)
	0.50	568 (459 - 677)	617 (508 - 726)	569 (193 - 946)	536 (483 - 588)	553 (511 - 594)	635 (607 - 662)	675 (655 - 696)
	0.75	549 (409 - 690)	735 (549 - 922)	834 (274 - 1394)	658 (581 - 735)	635 (586 - 684)	746 (703 - 790)	770 (742 - 797)
Sandbag Lunges	0.10	491 (375 - 607)	443 (298 - 589)	791 (32 - 1550)	441 (385 - 497)	463 (430 - 496)	524 (488 - 561)	566 (541 - 591)
	0.25	500 (415 - 584)	648 (475 - 821)	742 (292 - 1191)	516 (464 - 567)	529 (495 - 563)	607 (584 - 631)	686 (668 - 705)
	0.50	591 (499 - 684)	859 (725 - 993)	706 (402 - 1010)	592 (534 - 649)	587 (553 - 621)	706 (677 - 735)	811 (788 - 834)
	0.75	588 (454 - 721)	957 (761 - 1153)	783 (426 - 1141)	679 (627 - 732)	659 (615 - 703)	792 (753 - 830)	941 (932 - 951)
Wallballs	0.10	184 (25 - 344)	387 (243 - 530)	524 (-302 - 1350)	368 (305 - 431)	345 (299 - 390)	415 (381 - 449)	490 (454 - 526)
	0.25	282 (177 - 387)	486 (369 - 603)	580 (138 - 1022)	405 (350 - 459)	395 (352 - 437)	512 (481 - 542)	617 (588 - 646)
	0.50	432 (306 - 557)	579 (476 - 681)	702 (406 - 999)	469 (405 - 534)	482 (444 - 519)	609 (574 - 643)	764 (738 - 791)
	0.75	544 (383 - 705)	645 (542 - 748)	553 (315 - 792)	585 (509 - 660)	587 (534 - 640)	707 (661 - 753)	924 (889 - 959)

Supplementary Table S6: Mean ± SD of total race time and individual stations (absolute and relative) for the Top 100 distinct male PRO athletes per season (fastest performance per athlete per season).

Parameter		Season 1	Season 2	Season 3	Season 4	Season 5	Season 6	Season 7	ANOVA
Overall time	Absolute [min:sec]	70:43 ± 03:29	70:52 ± 04:40	93:36 ± 15:37	64:40 ± 03:12	61:13 ± 02:13	59:25 ± 01:34	57:15 ± 01:02	$F(6, 257.8) = 229.3$, $p < 0.001$; $\omega_p^2 = 0.84$
	Relative [% Total]	-	-	-	-	-	-	-	-
Running	Absolute [min:sec]	36:04 ± 02:18	35:19 ± 02:43	43:34 ± 06:00	32:58 ± 01:57	30:51 ± 01:27	30:17 ± 01:41	27:50 ± 01:54	$F(6, 327.6) = 215.2$, $p < 0.001$; $\omega_p^2 = 0.79$
	Relative [% Total]	51.0 ± 2.5	49.9 ± 2.9	47.0 ± 4.6	51.0 ± 2.0	50.4 ± 2.2	51.0 ± 2.9	48.6 ± 3.2	$F(6, 412.3) = 24.7$, $p < 0.001$; $\omega_p^2 = 0.25$
Ski-Erg	Absolute [min:sec]	04:00 ± 00:10	03:59 ± 00:11	04:15 ± 00:16	03:53 ± 00:09	03:49 ± 00:08	03:50 ± 00:08	03:49 ± 00:07	$F(6, 381.9) = 61.9$, $p < 0.001$; $\omega_p^2 = 0.48$
	Relative [% Total]	5.7 ± 0.3	5.6 ± 0.4	4.6 ± 0.6	6.0 ± 0.3	6.2 ± 0.2	6.4 ± 0.2	6.7 ± 0.2	$F(6, 522.9) = 360.2$, $p < 0.001$; $\omega_p^2 = 0.80$
Sled-Push	Absolute [min:sec]	02:22 ± 00:25	02:48 ± 01:10	06:15 ± 02:15	02:56 ± 00:53	02:50 ± 00:24	02:32 ± 00:21	02:28 ± 00:20	$F(6, 329.2) = 115.5$, $p < 0.001$; $\omega_p^2 = 0.67$
	Relative [% Total]	3.3 ± 0.6	3.9 ± 1.6	6.6 ± 1.6	4.5 ± 1.4	4.6 ± 0.6	4.3 ± 0.5	4.3 ± 0.6	$F(6, 418.6) = 70.8$, $p < 0.001$; $\omega_p^2 = 0.50$
Sled-Pull	Absolute [min:sec]	04:27 ± 00:39	05:20 ± 01:10	09:34 ± 06:22	04:02 ± 00:38	03:51 ± 00:31	03:43 ± 00:28	03:29 ± 00:31	$F(6, 244.1) = 54.3$, $p < 0.001$; $\omega_p^2 = 0.56$
	Relative [% Total]	6.3 ± 0.8	7.5 ± 1.5	9.9 ± 4.4	6.2 ± 0.9	6.3 ± 0.7	6.2 ± 0.7	6.1 ± 0.9	$F(6, 324.1) = 46.9$, $p < 0.001$; ω_p^2 2 = 0.45
Burpee Broad-Jump	Absolute [min:sec]	03:28 ± 00:36	03:24 ± 00:43	04:11 ± 01:13	02:49 ± 00:29	02:41 ± 00:25	02:45 ± 00:21	02:46 ± 00:16	$F(6, 366.1) = 52.6$, $p < 0.001$; $\omega_p^2 = 0.45$
	Relative [% Total]	4.9 ± 0.8	4.8 ± 0.9	4.5 ± 1.0	4.4 ± 0.7	4.4 ± 0.6	4.6 ± 0.6	4.8 ± 0.5	$F(6, 438.4) = 9.6$, $p < 0.001$; $\omega_p^2 = 0.10$
Rowing	Absolute [min:sec]	04:15 ± 00:12	04:16 ± 00:21	04:40 ± 00:23	04:08 ± 00:10	04:01 ± 00:09	04:01 ± 00:08	03:57 ± 00:06	$F(6, 338.4) = 70.1$, $p < 0.001$; $\omega_p^2 = 0.55$
	Relative [% Total]	6.0 ± 0.2	6.1 ± 0.6	5.1 ± 0.6	6.4 ± 0.3	6.6 ± 0.3	6.8 ± 0.2	6.9 ± 0.2	$F(6, 396.9) = 224.3$, $p < 0.001$; $\omega_p^2 = 0.77$
Farmers Carry	Absolute [min:sec]	01:48 ± 00:24	01:53 ± 00:20	02:21 ± 00:41	01:43 ± 00:15	01:39 ± 00:13	01:33 ± 00:09	01:26 ± 00:07	$F(6, 391.5) = 52.8$, $p < 0.001$; $\omega_p^2 = 0.44$
	Relative [% Total]	2.6 ± 0.5	2.6 ± 0.4	2.5 ± 0.5	2.7 ± 0.4	2.7 ± 0.3	2.6 ± 0.2	2.5 ± 0.2	$F(6, 508.8) = 3.5$, $p = 0.002$; $\omega_p^2 = 0.03$
Sandbag Lunges	Absolute [min:sec]	03:52 ± 00:33	03:52 ± 00:34	05:25 ± 01:33	03:38 ± 00:31	03:21 ± 00:22	03:12 ± 00:17	03:09 ± 00:16	$F(6, 332.4) = 79.3$, $p < 0.001$; $\omega_p^2 = 0.58$
	Relative [% Total]	5.5 ± 0.7	5.4 ± 0.7	5.8 ± 1.2	5.6 ± 0.7	5.5 ± 0.5	5.4 ± 0.4	5.5 ± 0.4	$F(6, 405.7) = 2.6$, $p = 0.017$; $\omega_p^2 = 0.02$
Wallballs	Absolute [min:sec]	06:11 ± 01:13	05:32 ± 01:04	06:37 ± 02:01	04:51 ± 00:47	04:31 ± 00:41	04:19 ± 00:34	03:57 ± 00:27	$F(6, 353.3) = 69.2$, $p < 0.001$; $\omega_p^2 = 0.53$
	Relative [% Total]	8.7 ± 1.6	7.8 ± 1.4	7.0 ± 1.4	7.5 ± 1.1	7.4 ± 1.0	7.2 ± 0.9	6.9 ± 0.7	$F(6, 417.1) = 27.8$, $p < 0.001$; $\omega_p^2 = 0.27$
Roxzone	Absolute [min:sec]	04:19 ± 01:00	04:36 ± 01:01	07:03 ± 02:44	03:47 ± 00:47	03:43 ± 00:54	04:25 ± 01:35	04:10 ± 01:02	$F(6, 502.8) = 56.9$, $p < 0.001$; $\omega_p^2 = 0.40$
	Relative [% Total]	6.1 ± 1.3	6.5 ± 1.3	7.5 ± 2.2	5.8 ± 1.1	6.1 ± 1.4	7.4 ± 2.7	7.3 ± 1.8	$F(6, 671.4) = 15.8$, $p < 0.001$; $\omega_p^2 = 0.12$

Supplementary Table S7: Mean ± SD of total race time and individual stations (absolute and relative) for the Top 100 distinct female PRO athletes per season (fastest performance per athlete per season).

Parameter		Season 1	Season 2	Season 3*	Season 4	Season 5	Season 6	Season 7	ANOVA
Overall time	Absolute [min:sec]	81:25 ± 06:13	80:05 ± 05:48	95:45 ± 15:59	73:16 ± 03:51	69:14 ± 03:14	66:39 ± 02:24	63:59 ± 01:31	$F(6, 355.2) = 202.6$, $p < 0.001$; $\omega_p^2 = 0.77$
	Relative [% Total]	-	-	-	-	-	-	-	-
Running	Absolute [min:sec]	40:53 ± 03:35	38:44 ± 03:10	45:24 ± 06:50	37:17 ± 02:03	35:16 ± 02:10	33:40 ± 02:18	30:38 ± 01:43	$F(6, 353.4) = 142.2$, $p < 0.001$; $\omega_p^2 = 0.70$
	Relative [% Total]	50.3 ± 3.0	48.4 ± 2.8	47.7 ± 4.0	50.9 ± 2.4	50.9 ± 2.1	50.5 ± 3.0	47.9 ± 2.4	$F(6, 511.9) = 23.4$, $p < 0.001$; $\omega_p^2 = 0.21$
Ski-Erg	Absolute [min:sec]	04:39 ± 00:15	04:39 ± 00:16	04:50 ± 00:19	04:27 ± 00:12	04:25 ± 00:11	04:26 ± 00:20	04:25 ± 00:09	$F(6, 575.8) = 27.0$, $p < 0.001$; $\omega_p^2 = 0.21$
	Relative [% Total]	5.7 ± 0.4	5.8 ± 0.4	5.1 ± 0.6	6.1 ± 0.3	6.4 ± 0.3	6.7 ± 0.5	6.9 ± 0.2	$F(6, 563.4) = 147.5$, $p < 0.001$; $\omega_p^2 = 0.61$
Sled-Push	Absolute [min:sec]	02:49 ± 00:37	03:54 ± 01:11	06:32 ± 02:34	03:29 ± 01:00	03:06 ± 00:31	02:50 ± 00:27	02:50 ± 00:31	$F(6, 539.9) = 79.8$, $p < 0.001$; $\omega_p^2 = 0.46$
	Relative [% Total]	3.5 ± 0.7	4.9 ± 1.4	6.8 ± 2.1	4.8 ± 1.3	4.5 ± 0.7	4.2 ± 0.7	4.4 ± 0.8	$F(6, 565.7) = 41.8$, $p < 0.001$; $\omega_p^2 = 0.30$
Sled-Pull	Absolute [min:sec]	05:43 ± 01:27	06:45 ± 01:36	07:08 ± 02:18	04:42 ± 00:55	04:23 ± 00:33	04:10 ± 00:36	04:04 ± 00:27	$F(6, 470.9) = 95.4$, $p < 0.001$; $\omega_p^2 = 0.54$
	Relative [% Total]	7.0 ± 1.6	8.4 ± 1.8	7.3 ± 1.3	6.4 ± 1.1	6.3 ± 0.7	6.3 ± 0.8	6.3 ± 0.6	$F(6, 504.7) = 48.3$, $p < 0.001$; $\omega_p^2 = 0.36$
Burpee Broad-Jump	Absolute [min:sec]	04:26 ± 00:52	04:04 ± 00:53	04:52 ± 01:17	03:36 ± 00:42	03:29 ± 00:32	03:33 ± 00:28	03:31 ± 00:31	$F(6, 408.9) = 30.8$, $p < 0.001$; $\omega_p^2 = 0.30$
	Relative [% Total]	5.4 ± 0.9	5.1 ± 1.0	5.0 ± 0.8	4.9 ± 0.9	5.0 ± 0.7	5.3 ± 0.6	5.5 ± 0.8	$F(6, 441.4) = 9.3$, $p < 0.001$; $\omega_p^2 = 0.10$
Rowing	Absolute [min:sec]	04:51 ± 00:15	04:52 ± 00:17	05:12 ± 00:26	04:41 ± 00:12	04:33 ± 00:10	04:34 ± 00:09	04:31 ± 00:08	$F(6, 421.2) = 70.4$, $p < 0.001$; $\omega_p^2 = 0.49$
	Relative [% Total]	6.0 ± 0.4	6.1 ± 0.4	5.5 ± 0.5	6.4 ± 0.3	6.6 ± 0.3	6.9 ± 0.3	7.1 ± 0.2	$F(6, 497.6) = 138.5$, $p < 0.001$; $\omega_p^2 = 0.62$
Farmers Carry	Absolute [min:sec]	02:17 ± 00:32	02:20 ± 00:31	02:46 ± 00:46	02:06 ± 00:18	02:01 ± 00:17	01:51 ± 00:15	01:46 ± 00:12	$F(6, 408.4) = 42.1$, $p < 0.001$; $\omega_p^2 = 0.37$
	Relative [% Total]	2.8 ± 0.6	2.9 ± 0.5	2.9 ± 0.5	2.9 ± 0.4	2.9 ± 0.4	2.8 ± 0.3	2.8 ± 0.3	$F(6, 449.6) = 3.6$, $p = 0.002$; $\omega_p^2 = 0.03$
Sandbag Lunges	Absolute [min:sec]	04:25 ± 00:42	04:12 ± 00:36	05:28 ± 01:04	03:57 ± 00:36	03:41 ± 00:25	03:35 ± 00:19	03:27 ± 00:18	$F(6, 426.4) = 74.5$, $p < 0.001$; $\omega_p^2 = 0.50$
	Relative [% Total]	5.4 ± 0.7	5.2 ± 0.6	5.7 ± 0.7	5.4 ± 0.7	5.3 ± 0.5	5.4 ± 0.4	5.4 ± 0.5	$F(6, 459.3) = 4.4$, $p < 0.001$; $\omega_p^2 = 0.04$
Wallballs	Absolute [min:sec]	06:28 ± 01:39	05:28 ± 01:04	06:53 ± 02:35	04:44 ± 00:58	04:23 ± 00:41	04:11 ± 00:37	04:02 ± 00:32	$F(6, 346.7) = 64.1$, $p < 0.001$; $\omega_p^2 = 0.52$
	Relative [% Total]	7.9 ± 1.7	6.8 ± 1.2	7.1 ± 1.7	6.4 ± 1.2	6.3 ± 0.9	6.3 ± 0.9	6.3 ± 0.8	$F(6, 412.6) = 23.4$, $p < 0.001$; $\omega_p^2 = 0.24$
Roxzone	Absolute [min:sec]	04:58 ± 01:11	05:11 ± 01:06	06:56 ± 02:17	04:21 ± 00:54	04:03 ± 00:53	04:29 ± 01:32	04:39 ± 00:59	$F(6, 604.7) = 27.6$, $p < 0.001$; $\omega_p^2 = 0.21$
	Relative [% Total]	6.1 ± 1.3	6.5 ± 1.3	7.1 ± 1.7	5.9 ± 1.1	5.9 ± 1.3	6.7 ± 2.2	7.3 ± 1.5	$F(6, 611.8) = 12.6$, $p < 0.001$; $\omega_p^2 = 0.10$

*for Season 3 only 31 distinct athletes could be included due to a limited number of races in this season.
